# Interactive spatial scale effects on species distribution modeling: The case of the giant panda

**DOI:** 10.1038/s41598-019-50953-z

**Published:** 2019-10-10

**Authors:** Thomas Connor, Andrés Viña, Julie A. Winkler, Vanessa Hull, Ying Tang, Ashton Shortridge, Hongbo Yang, Zhiqiang Zhao, Fang Wang, Jindong Zhang, Zejun Zhang, Caiquan Zhou, Wenke Bai, Jianguo Liu

**Affiliations:** 1 Center for Systems Integration and Sustainability, Department of Fisheries and Wildlife, Michigan State University, East Lansing, MI USA; 2 Department of Geography, University of North Carolina, Chapel Hill, NC USA; 3 Department of Geography, Environment, and Spatial Sciences, Michigan State University, East Lansing, MI USA; 4 Department of Wildlife Ecology and Conservation, University of Florida, Gainesville, FL USA; 5 Key Laboratory of Southwest China Wildlife Resources Conservation, China West Normal University, Ministry of Education, Nanchong, China

**Keywords:** Ecological modelling, Biogeography

## Abstract

Research has shown that varying spatial scale through the selection of the total extent of investigation and the grain size of environmental predictor variables has effects on species distribution model (SDM) results and accuracy, but there has been minimal investigation into the interactive effects of extent and grain. To do this, we used a consistently sampled range-wide dataset of giant panda occurrence across southwest China and modeled their habitat and distribution at 4 extents and 7 grain sizes. We found that increasing grain size reduced model accuracy at the smallest extent, but that increasing extent negated this effect. Increasing extent also generally increased model accuracy, but the models built at the second-largest (mountain range) extent were more accurate than those built at the largest, geographic range-wide extent. When predicting habitat suitability in the smallest nested extents (50 km^2^), we found that the models built at the next-largest extent (500 km^2^) were more accurate than the smallest-extent models but that further increases in extent resulted in large decreases in accuracy. Overall, this study highlights the impacts of the selection of spatial scale when evaluating species’ habitat and distributions, and we suggest more explicit investigations of scale effects in future modeling efforts.

## Introduction

Spatial scale has long been recognized as important to consider in ecological research^1^. It varies along two main dimensions - grain, the spatial size of individual samples, and extent, the total area under study^2^. Because changes in the spatial scale of a study may result in complex effects that vary by species and system^3,4^, it is an important factor to consider when investigating habitat and species distributions^5^. Empirical investigations of the effects of spatial scale are not only rare, but also mainly involve varying the “local extent” surrounding a species or individual location within which environmental data are averaged^4,6^. While this may constitute an effective way to study the effects of spatial scale on species distributions and habitat selection, the effect of varying the local extent itself is constrained by both the grain size of the included environmental variables and the total extent of the study area. It has also been commonplace to select the grain size of a study a *priori* and without any kind of optimization, usually based on expert opinion and/or the format of the available data needed for modeling^7,8^.

One of the main reasons behind the usage of varying spatial scales between studies is that the goals of habitat modeling for a species can vary widely, ranging from investigations on the within-home range habitat selection of individual animals^9^ to the delineation of a species’ entire distribution^10^. These goals directly relate with Johnson’s (1980) four orders of habitat selection, which in descending order include geographic range, home range, differential space use within the home range, and the final procurement of resources^11^. Investigations into these different orders of habitat selection largely drive the spatial scale used in a given study. These are most apparent through changes to the total extent of analysis (a home range compared to a species’ geographic range), but they also influence the grain through the common use of coarser climate variables^8^ and lack of computational power for fine grains in studies of fourth-order (geographic range) processes^12^. Recent work to integrate multiple orders of habitat selection simultaneously through varying the local extent of included environmental predictor data and hierarchically modeling selection has shown that species may respond to environmental variables differently between orders and/or between scales within orders^4,13^.

**Figure 1:**
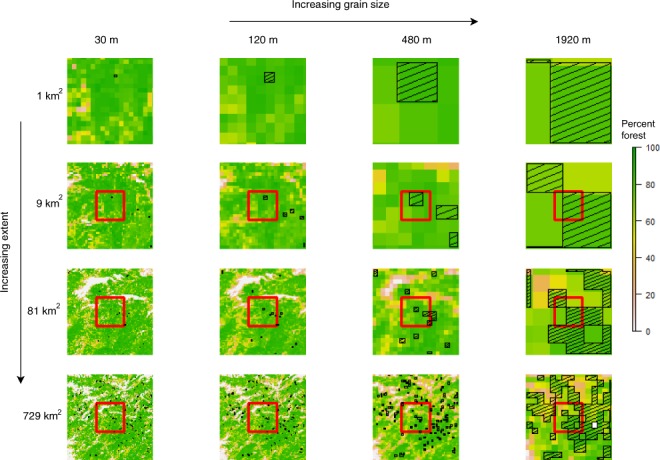
Visual representation of increasing spatial scale along two main axes – total extent and grain size. Percent forest is characterized at 4 extents and 4 grain sizes in an area around the middle of the giant panda range, with giant panda presence cells depicted with a hatch pattern. The red squares in rows 2 to 4 indicate the previous total extent shown in the previous row. Note: these are not the total extents examined in this study, they are purely a visual demonstration of the effects of changing spatial scale on environmental and presence data.

Another key driver of the scale of analysis in habitat and species distribution modeling is data availability. The grain size influences which environmental predictor variables can be included in modeling efforts. In general, data availability, such as the accessibility of climate observations and simulations of future climate^14,15^ is greater at larger grain sizes that become relevant at larger extents. The varying resolution of available datasets often results in the spatial aggregation of variables with smaller grain sizes to match the coarser scales of the other variables, or the interpolation of variables of larger grain sizes to match the finer resolution of the other variables^7^, with the latter approach increasing their spatial autocorrelation^16^. In addition to impacts on environmental variables, spatial aggregation to coarse scales can also affect the presence dataset used in the habitat modeling of the species in question (Fig. 1). For instance, data collected at a fine scale with points falling within 30 meters of one another may fall in different 30 m cells but the same aggregated 60 m cell. Potential impacts are exacerbated when cells are only defined as presence or absence, ignoring the number of points within a cell, the default option for the popular MaxEnt software^17^ and any species distribution model for which the species input is binary (presence or absence for a given location). The potential tradeoffs in model performance that occur with the aggregation of some variables to allow for the addition of others, and how this interacts with changes in the extent of analysis, is another scale-related topic that has been understudied.

While there have been empirical studies evaluating the effects of grain size^18–21^ and total study extent^22,23^ on habitat and species distribution modeling, little research has looked at the interactive effects of these components. Seo *et al*. (2009) examined the effects of grain size on distribution models of species with different range sizes (i.e., total extents), although they did not vary the total extent of analysis within a given species^24^. They found that model results of species with intermediate range sizes were more sensitive to changes in grain size compared to species with large or small ranges. Although Trivedi *et al*. (2008) built models at multiple grains and total extents, the sampling designs of the datasets used in the analysis varied for the different scales making direct comparisons difficult^25^. Khosravi *et al*. (2016) varied the grain size of their environmental predictors in addition to the local extent around each presence and background cell within which they averaged those predictors in modeling goitered gazelle distributions and found intermediately-sized grains and local extents to be most accurate, but they did not vary the total extent of the area modeled^26^. To the best of our knowledge, only one previous study varied both grain size and total extent, in which only two coarse grains (1 km and 10 km) and two broad extents (Africa and West Africa) were used. The smaller 1-km grain size at the regional (as opposed to continental) extent was found to best capture the distributions of three widespread African species (Patas monkey, bull frog, and rock hyrax) at the edge of their range^27^. A deeper analysis of the interactive effects of the total extent and grain size of environmental variables on habitat and species distribution modeling is crucial, since the range of scales used to model habitat or species distributions can vary considerably (e.g. total extents of 40 km^2^ to 66,456 km^2^ and grain sizes of 30 m to 1,000 m for giant pandas^9,10,28^), with potentially important effects on model accuracy and sensitivity.

To address these knowledge gaps, here we evaluate the effects of simultaneously changing the grain size and total extent, as well as the use of different environmental predictor variables, on habitat suitability and species distribution modeling of a demonstration species, the giant panda (*Ailuropoda melanoleuca*). Due to their elusive nature and protected status, there are still many uncertainties about giant panda behavior in the wild^29^. Although there have been numerous studies on giant panda habitat selection and distribution, there are still many discrepancies concerning the relative importance of and responses to different environmental variables used to model their habitat^30^. Like most species, there has also been little investigation into spatial scale effects, which may account for some discrepancies seen in the literature. Giant pandas also serve as an effective case study to test for interactive spatial scale effects on habitat and distribution modeling, particularly of habitat specialist species, due to their dependence on understory bamboo and sensitivity to human disturbances^29,31^. Any evidence of interactive scale effects on model performance, as well as scale interactions with environmental variable selection, will have important implications for both giant panda research and species distribution modeling in general and will imply that spatial scale needs to be explicitly considered for effective research and conservation.

## Methods

### Study area and extents

**Figure 2:**
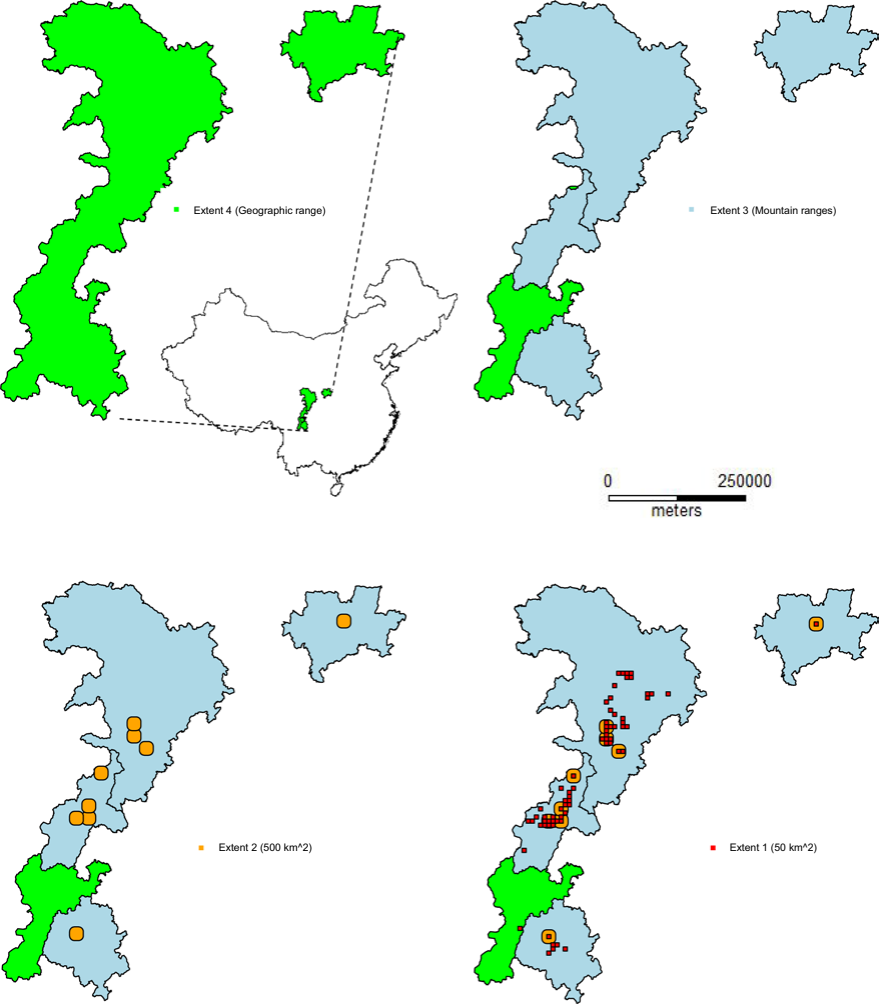
Total extents used in the analysis of scale effects on modeling giant panda habitat suitability and distribution. The smallest total extent was replicated 68 times, total extent 2 was replicated nine times, and total extent 3 was replicated four times across the current panda geographic range, which served as total extent 4.

We chose four different total extent sizes to model panda distributions consisting of 50 km^2^, 500 km^2^, the mountain ranges containing these extents (mean area of 18,264 km^2^) and the entire geographic panda range of approximately 109,585 km^2^ (Table 1, Fig. 2). The nested structure of these extents allowed for replication of the smaller three within the largest. Specifically, the total geographic range was gridded into 50 km^2^ blocks, and those with at least 20 panda presence locations were selected for modeling (n = 68). Several of these were then randomly selected to be buffered so that there was n = 9 non-overlapping total extents of 500 km^2^. Finally, distinct mountain ranges that contained one or more of these 500 km^2^ total extents were selected as replicates of the third total extent (n = 4).

**Table 1:** The number of presence cells and their prevalence (presence cells divided by the total cells used) at each extent and grain size.

Total extent	Total extent 1 (50 km^2^)		Total extent 2 (500 km^2^)		Total extent 3 (Mountain ranges, mean 18,264 km^2^		Total extent 4 (Geographic range, 109,585 km^2^)	
Grain size (m)	Mean presence cells (SD)	Mean Prevalence (SD)	Mean presence cells (SD)	Mean Prevalence (SD)	Mean presence cells (SD)	Mean Prevalence (SD)	Mean presence cells (SD)	Mean Prevalence (SD)
30	30.5 (11.3)	5.5E-04 (2.0E-03)	154.4 (77.8)	2.5E-04 (1.2E-04)	1137.6 (845.1)	3.3E-05 (2.4E-05)	4707 (129.0)	9.3E-06 (2.5E-07)
60	25.4 (6.1)	1.8E-03 (4.0E-04)	153.7 (77.3)	9.7E-04 (4.9E-04)	1130.9 (837.9)	1.3E-04 (9.6E-05)	4679 (128.1)	3.7E-05 (1.0E-06)
120	24.8 (5.9)	7.1E-03 (1.7E-03)	151.1 (75.8)	3.9E-03 (2.0E-03)	1113.1 (822.9)	5.1E-04 (3.8E-04)	4613.8 (126.2)	1.5E-04 (4.0E-06)
240	22.9 (5.7)	0.03 (6.5E-03)	144.3 (71.6)	0.01 (7.3E-03)	1065.7 (776.5)	2.0E-03 (1.4E-03)	4423.3 (109.7)	5.6E-04 (1.4E-05)
480	19.8 (5.1)	0.09 (0.02)	127.9 (60.5)	0.05 (0.02)	952.1 (676.0)	7.0E-03 (5.0E-03)	3975.3 (85.0)	2.0E-03 (4.3E-05)
960	14.5 (4.0)	0.26 (0.07)	98.7 (43.9)	0.16 (0.07)	753.3 (509.8)	0.02 (0.01)	3159.3 (57.9)	6.4E-03 (1.2E-04)
1920	8.1 (2.9)	0.67 (0.24)	62.3 (23.8)	0.40 (0.15)	516.7 (323.1)	0.06 (0.04)	2172 (29.1)	0.02 (2.4E-04)

### Environmental variables and grain sizes

We chose environmental predictors based on their biological significance for pandas, as well as data availability. Our base model included elevation, slope, percent forest, distance to a main road, and distance to a stream or river (Table 2). Main road and stream/river data were obtained at a scale of 1:250000 from the Chinese National Fundamental Geographic Information Database, maintained by the National Geomatics Center of China^32^. The smallest available grain size for elevation was a digital elevation model (DEM) of 30 m, obtained through NASA’s SRTM mission^33^. Slope was derived from this DEM in ArcGIS 10.4. Percent forest was obtained from the global dataset developed by Hansen *et al*. (2013) at a grain size of 27 m^34^. These data were resampled to 30 m using bilinear interpolation, while the distance variables were calculated using a grain size of 30 m. We then successively spatially aggregated these variables by a factor of 2 using the mean of the smaller cells to develop 6 additional sets of predictors of increasing grain size. We also did the analyses using the median value of the smaller cells, which had nearly identical results. For each of the 4 extents, we thus included the same environmental variables at 7 different grain sizes ranging from 30 to 1920 m (Table 2).

**Table 2:** Summary of environmental variables and grain sizes used in the study.

Variable name (unit)	Grain sizes (m)
Elevation (m)	30, 60, 120, 240, 480, 960, 1920
Slope (degrees)	30, 60, 120, 240, 480, 960, 1920
Percent forest (%)	30, 60, 120, 240, 480, 960, 1920
Distance to main road (m)	30, 60, 120, 240, 480, 960, 1920
Distance to stream (m)	30, 60, 120, 240, 480, 960, 1920
MODIS phenology metrics^*^	240, 480, 960, 1920
Annual mean temperature (°C * 10)	960, 1920
Temperature seasonality (standard deviation * 100)	960, 1920
Temperature annual range (max temp – min temp)	960, 1920
Annual precipitation (mm)	960, 1920
Precipitation seasonality (coefficient of variation)	960, 1920

*MODIS phenology metrics used in the models were reduced to five principal components using PCA.

At the grain size of 240 m, we also built models that included 11 MODIS-derived land surface phenology metrics that have been used to accurately predict bamboo^35^ and giant panda^36^ distributions. These 11 metrics describe the timing and magnitude of phenophases of the land surface^35^. Many of these metrics are highly correlated (r > 0.9). To avoid multi-collinearity issues^37^, we conducted a principal component analysis (PCA) on the 11 metrics and used the first five components (explaining over 99% of the variation) as environmental variables in our models. We resampled these first five components from native MODIS resolution of 250 m to the grain size of the coarsened base variables at 240 m using bilinear interpolation. We then aggregated this new set of environmental variables in the same manner as before to derive 3 additional sets of variables at 480, 960, and 1920 m (Table 2). The MODIS-derived phenology variables were averaged from satellite data acquired in 2000–2002, the main sampling years of the 3^rd^ National Giant Panda Survey^38^.

Finally, for the habitat modeling at the 960 and 1920 m grain sizes, we trained models that additionally incorporated remotely sensed climate variables. The source of the climate information was the Deblauwe *et al*. (2016) dataset of bioclimatic variables derived from remotely-sensed measurements with a ~6 km resolution^39^. Tang *et al*.^15^ later interpolated these measurements to a 1 km resolution using an inverse weighted distance approach, which we also used. Five bioclimatic variables were selected based on their ecological meaning for giant pandas and lack of collinearity^15^. These included annual mean temperature, temperature seasonality, temperature annual range, annual precipitation, and precipitation seasonality. We resampled the climate data using bilinear interpolation to match the 960 m grain size, and once again aggregated by a factor of 2 using a mean function to produce the final set of environmental variables at 1920 m (Table 2). Like the phenology variables, the satellite-derived bioclimatic variables were averaged between the years of 2000 and 2002 to capture the conditions found during the 3^rd^ National Giant Panda Survey.

### Giant panda presence data

To model giant panda habitat at different scales, we used presence data from the 3^rd^ National Giant Panda Survey^38^. These data were collected between 2000 and 2003 across the current giant panda geographic range and consist of georeferenced locations that had signs of giant panda presence (e.g., feces, hair; State Forestry Administration 2006). The sampling technique involved placing line transects within 2 km^2^ grid cells across the entire known panda range and searching for panda signs along these transects. For the 30 m grain size, the smallest extent replicates had a mean of 30.5 (SD = 11.3) presence cells, replicates for the second extent level had a mean of 154.4 (SD = 77.8) presence cells, replicates for the third level had a mean of 1137.6 (SD = 845.1) presence cells, and the total range (extent 4) had 4707 presence cells (Table 1).

### Habitat suitability modeling

Because only giant panda presence data were available for this analysis, we ran MaxEnt^17^ models for the various sets of predictor variables at different grain sizes and total extents. MaxEnt makes use of machine learning techniques to derive a probability distribution of environmental suitability across a landscape from the environmental predictor variables^17^. The algorithm attempts to minimize the relative entropy in the predicted suitability between presence locations and background locations^40^. We used 10,000 randomly sampled points from within the precise polygon for a given total extent to serve as background locations to the models. At the smaller total extents, limited cells in the larger grain sizes resulted in all of the raster cells of the environmental predictors serving as background data. For example, the models built at the smallest total extent and with the 1920 m grain size only had a total of 12 cells. We selected background points only from areas in which a panda could feasibly occur^41^, which we defined as below 3600 m in elevation due to the lack of bamboo in alpine areas. We used a k-fold partitioning design to develop five different random combinations of training (80%) and testing (20%) data for model input at every grain size and extent.

To evaluate each model, we calculated the Area under the Receiving Operating Characteristic Curve (AUC), which measures the ability to discriminate between observed presence and background grid cells. Due to criticism of AUC as an accuracy metric that may result in inflated and spurious values^42,43^, we also calculated True Skill Statistics (TSS) and correlation coefficients between the predicted suitability values at test presences vs. background locations obtained from each model (hereafter referred to as ‘correlation coefficient’). TSS is advantageous in our case due to its independence from prevalence^44^ (which changes across extent and grain, Table 1), and we calculated it at the threshold for conversion of the predicted probability surface into a binary presence and absence surface that maximized its value (i.e., maximum TSS).

Replication of the smaller total extents allowed us to examine the effect of a given study area’s placement on the environmental gradients (e.g., latitude, percent forest) found across the panda range, and if this modified the effect of total extent and/or grain. We calculated the mean value of every environmental predictor within every total extent replicate to determine that extent’s placement on the environmental gradient for each respective predictor. In a similar manner, we were able to determine if the amount of environmental heterogeneity (e.g., elevation, percent forest) in a given study area had an impact on the effect of total extent/grain size on model accuracy. We calculated the standard deviation of every environmental predictor within every study area to represent the environmental heterogeneity for a given predictor in each respective total extent replicate.

We also evaluated the performance of models built at larger total extents in predicting habitat suitability in the smallest total extent nested within those larger total extents. This was done for the nine smallest extent replicates that were buffered to produce the next total extent. This allowed us to determine the relationship between increasing total extent and predictive accuracy using the same test area and data in each case. We also produced predicted habitat suitability maps to visually compare the results of each model. This analysis was done using the base models at the 30-m grain size.

To further evaluate the tradeoffs associated with the addition of the MODIS and climate variables at the larger grain sizes, we compared model parsimony using Akaike’s Information Criterion^45^ corrected for small sample sizes (AICc). This measure is particularly important when evaluating models with a different number of variables, as it incorporates a penalty for additional explanatory variables and overfitting the model^46^. AICc cannot effectively evaluate between models of different total extent or grain size, however, because changing either of these components of spatial scale changes the sample locations included in the model, thus making the information criterion uncomparable^47^.

To evaluate the effects of environmental predictors on the various models at different scales, we conducted permutation importance tests^17^. The permutation test slightly changes the values of each environmental variable among the training presence and background points, and measures the loss in AUC through this process. This was done for each variable separately, and the final values were normalized to percentages. We also produced environmental response plots for each variable to visualize their effects on habitat suitability and how these effects changed across scales, suites of variables, and study areas.

In summary, base models were built at seven different grain sizes, base + phenology models were built at four different grain sizes, and base + phenology + climate models were built at two different grain sizes. These models were built within all replicates of the four total extents. All reported statistics (e.g., AUC, TSS, AICc) are a result of averaging the five training/testing model runs. All these analyses utilized the ‘dismo’ package in the R environment^48,49^.

## Results

**Figure 3:**
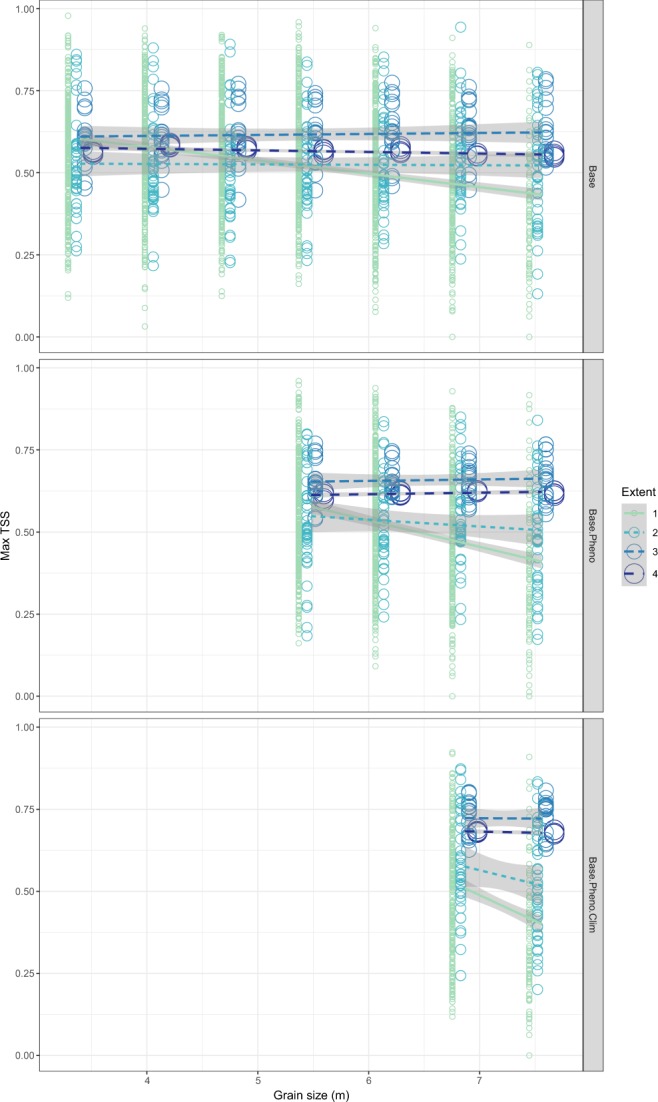
The effect of total extent, grain size, and variable combination on model accuracy (measured by the maximum True Skill Statistic (TSS)). Different variable combinations are presented in different panels - “Base” models include elevation, percent forest, slope, distance to main road, and distance to stream variables, “Base.Pheno” models include these five variables plus five phenology variables derived from MODIS data and PCA, and “Base.Pheno.Clim” models include all of these variables in addition to the five bioclimatic variables listed in Table 2.

**Figure 4:**
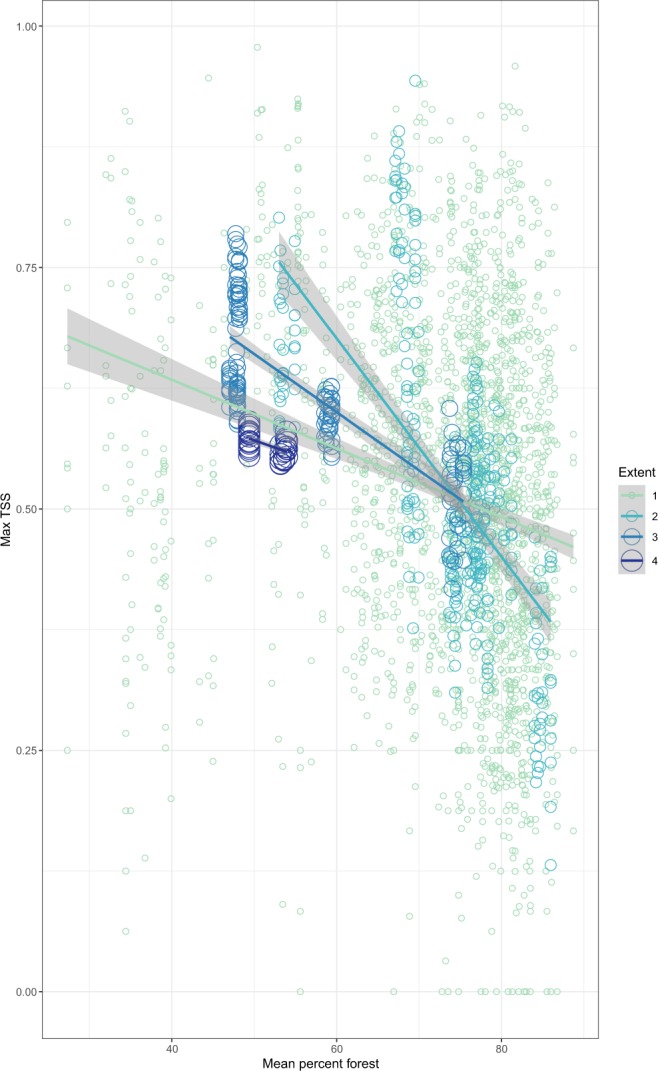
The effect of the position of a study area along an environmental gradient (percent forest cover) on model accuracy, as well as its impact on the effect of total extent on model accuracy. Evaluation scores of models built at different total extents are represented by different colors and differently sized circles.

The accuracy of the models generally increased with increasing total extent and the addition of environmental variables at coarser grains. Increasing grain size in the smallest total extent decreased accuracy, as measured by the max TSS, but this effect was lost in the three larger total extents (Fig. 3). The AUC and correlation coefficient results mirrored the max TSS except that increasing grain size reduced accuracy in total extent 2 in addition to the smallest extent (Fig. S1). The placement of a given study area along some environmental gradients had a strong effect on accuracy, and this placement also moderated the effect of total extent on accuracy. For example, the accuracy of models using the base set of variables generally decreased with increasing forest cover, but this decrease occurred at different rates across the different total extents so that their accuracies converged at about 75% forest cover (Fig. 4). A similar pattern emerged with increasing latitude in extents 2 and 3, but little to no effect was seen across the elevation and mean annual temperature gradients (Fig. S2). Clear effects of grain size on model accuracy across environmental gradients were not evident, as changes in accuracy showed nearly the same trend across all grain sizes (Fig. S3). Increasing environmental heterogeneity within a given study area resulted in increased model accuracy, but did not appear to moderate the effects of either total extent or grain size (except for the 1920-m grain) (Figs S4 and S5).

**Figure 5:**
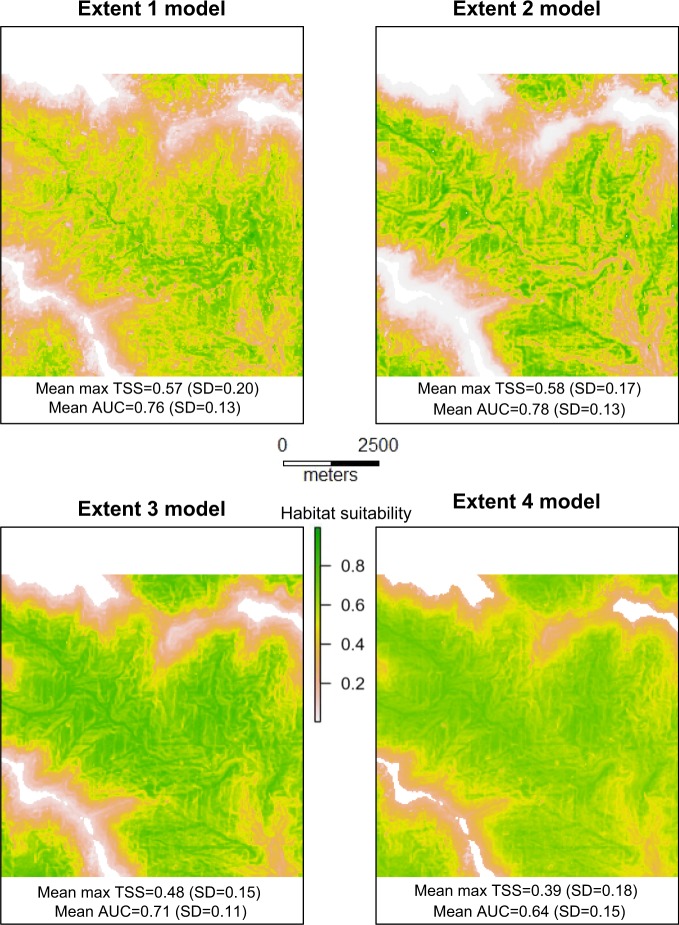
Giant panda habitat suitability predictions of one of the study area replicates of the smallest extent produced by models using the base variables at the 30-m grain size and trained at increasingly larger extents. Accuracy statistics for models trained at a given total extent are reported under the predicted suitability maps produced by that respective model.

The models built at different total extents resulted in different suitability predictions of the smallest (total extent 1) study areas nested within them (Fig. 5). The second-smallest total extent models had the highest accuracy when predicted to smallest total extent area (mean max TSS = 0.58 (SD = 0.17), mean AUC = 0.78 (SD = 0.13), followed by the smallest extent models (mean max TSS = 0.57 (SD = 0.20), mean AUC = 0.76 (SD = 0.13), the second-largest total extent models (mean max TSS = 0.48 (SD = 0.15), mean AUC = 0.71 (SD = 0.11), and finally the largest total extent models (mean max TSS = 0.39 (SD = 0.18), mean AUC = 0.64 (SD = 0.15)). The predicted suitability maps of these models were qualitatively very different, with the most accurate models producing predictions that had a more pronounced difference between high and low suitability areas while the largest total extent models produced predictions with a “smoothed” appearance (Fig. 5).

Increasing grain size in order to incorporate the additional phenology and climate variable sets at the 240 m and 960 m grains, respectively, resulted in improved model accuracy in the larger two total extent sizes but had no significant effect at a 95% confidence interval in the smaller two total extent sizes (Fig. 3). The AICc rankings generally agreed with the accuracy metrics, with the addition of phenology and climate variables resulting in higher ranked models at the two largest total extents, but generally lower ranked models across the replicates of the two smallest total extents (Table 3).

**Table 3:** Comparisons of parsimony between models containing different variable sets built at increasing extent.

Comparison	Total extent 1			Total extent 2			Total extent 3			Total extent 4		
	−	+	No change	−	+	No change	−	+	No change	−	+	No change
Base: Base + Phenology	0.63	0.23	0.14	0.84	0.14	0.02	0.36	0.64	0	0	1	0
Base + Phenology: Base + Phenology + Climate	0.43	0.36	0.21	0.69	0.29	0.02	0.08	0.93	0	0	1	0
Base: Base + Phenology + Climate	0.53	0.29	0.18	0.84	0.13	0.02	0.05	0.95	0	0	1	0

Proportions of model replicates that featured increased AICc values of greater than 2 and thus reduced parsimony (−), proportions of replicates that featured decreased AICc values of greater than 2 and thus improved parsimony (+), and proportions of replicates that featured changes in AICc values less than 2 and thus no change in parsimony (0) are presented.

**Figure 6:**
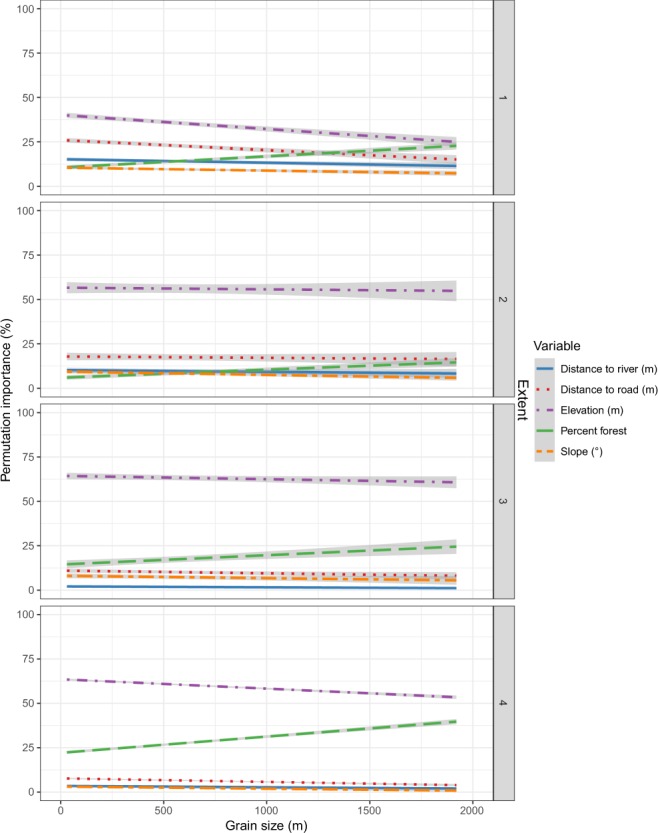
Variable permutation importance values (in percent) for the variables included in the MaxEnt habitat suitability models incorporating the base variable set. Grain size is plotted on the x-axis and models built at the different total extents are separated by panel.

The contribution of individual environmental variables to the models, as measured by the permutation importance tests, varied with total extent and grain. For example, although elevation was important to the models incorporating the base variables at the smallest total extent, with an average of about 40% permutation importance, this increased to about 60% permutation importance for the base models at three larger total extents (Fig. 6). Conversely, the distance to road variable lost importance as total extent increased with average values of 22, 17, 10, and 7% importance in base models trained at smallest to largest total extents, respectively. Similar losses occurred in slope and distance to river variables with increasing total extent. Though the permutation importance of most variables did not change much with increasing grain size, elevation tended to lose importance and percent forest tended to gain importance (Fig. 6). Elevation also lost importance with the incorporation of phenology and climate variable sets at the 240 and 960 m grain sizes, respectively (Fig. S6). Mean annual temperature proved particularly important to the models built at the second-largest (mean permutation importance of 17%) and largest (permutation importance of 33%) total extents.

**Figure 7:**
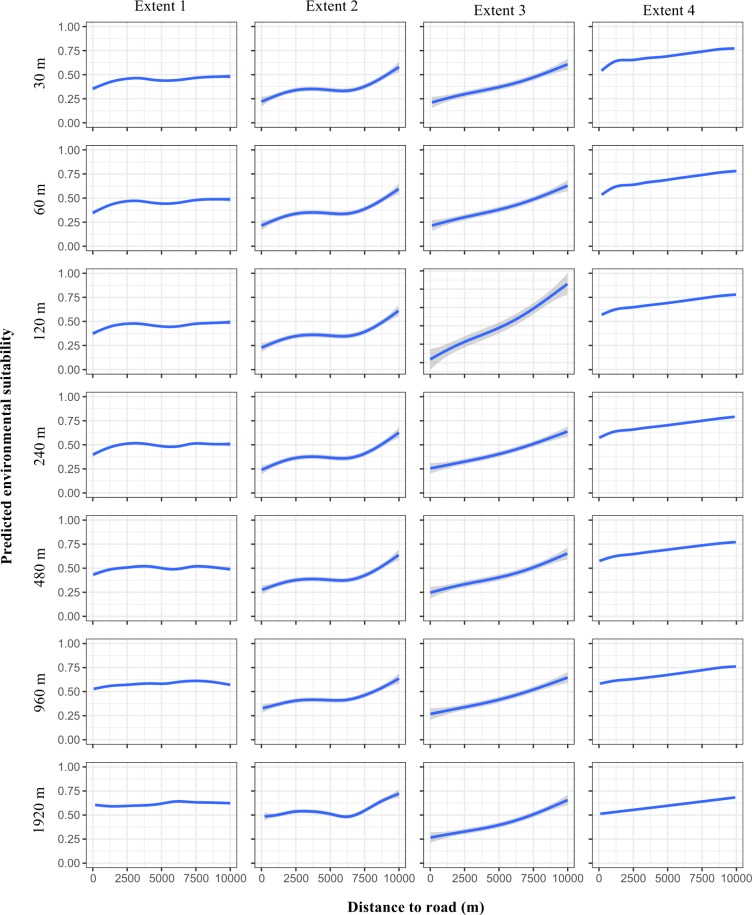
Responses of the base model predictions of giant panda habitat suitability in one of the sampled study areas to changes in the distance to road variable at increasing (left to right) total extent and increasing (top to bottom) grain size.

Predicted suitability responses to the environmental variables changed considerably across the replicated study areas, and to a lesser extent due to changes in scale. For example, although there was generally a positive response to forest cover across the replicates of the smallest total extent, the magnitude of the effect varied and in some areas there was zero effect (Fig. S7). Many of the models built in the replicates of the smallest total extent featured different environmental response curves than the models built in the larger total extents in which they were nested, while those models built at the larger total extents featured similar environmental responses to each other. The effect of the distance to road variable on environmental suitability is a good example of this, with increasing distance to roads resulting in clear positive effects in the models built at larger total extents but varied effects in the models at the smallest total extent (Figs 7 and S8). Increasing grain size tended to result in losses in detail in the environmental responses, with some variables having little to no effect on suitability at the 1920-m grain size (Fig. 7).

## Discussion

Our results highlight the interactive and potentially large effects of spatial scale on species distribution modeling. Unlike previous studies, ours is the first to use a single, comprehensive presence dataset to investigate these effects along both main dimensions of spatial scale – total extent and grain size. Models built and validated at larger total extents generally had higher accuracies, likely because as total extent increases, so does the amount of presence data and the range of the environmental variable values used to model habitat. More presence data allows the models to better approximate the response of a species to the environment, though MaxEnt has been shown to produce accurate predictions at low sample sizes^50,51^. Perhaps more important is the fact that, at larger total extents, a larger range of environmental predictor values allows the models to better differentiate between areas of low and high suitability at that given total extent^22^.

A potential exception to these relationships is a situation in which a species’ response to its environment changes significantly across its geographic range^52^. In this case, a single model with fixed parameters may not be appropriate to describe the responses of individuals to their environment in different populations across their geographic distribution. Our results confirm this phenomenon for the giant panda, as the models trained in the replicates of the second-largest total extent (representing individual mountain ranges) had higher accuracies than models trained at the entire geographic range. Because there is little to no dispersal among giant panda populations living in several of the different mountain ranges due to natural (e.g., large rivers) and anthropogenic (e.g., highways) barriers^53^, these populations have likely adapted to specific environmental conditions found in their respective mountain ranges. This suggests that the benefits associated with modeling at larger total extents (more presence locations, increased range of environmental variables) do not necessarily outweigh the negative effects associated with including populations with different environmental responses within the same model. Therefore, this effect should be tested in any geographic range-wide evaluation and depending on the results, predictions from models trained at a mosaic of total extents should be used as opposed to a single, geographic range-wide model. Alternatively, modeling techniques that allow for non-stationarity in environmental responses could be considered^52^.

Although grain size had a strong effect on model accuracy in models built at the smallest total extent with smaller grain sizes producing more accurate predictions, this effect was negated by increasing the total extent. Models built at the smallest total extent of 50 km^2^ were likely negatively affected by increasing grain because of the severe loss of presence and background cells with each aggregation in grain. By the largest grain size, there was only an average of 8 presence cells and 12 total background cells per 50 km^2^ study area. This resulted in large overlaps between presence and background environmental conditions and a weak signal between suitable and unsuitable habitat^54^. Our results suggest giant panda distributions at total extents of 500 km^2^ and higher can be accurately modeled using grain sizes up to 1920 m, however.

The method of aggregation could also impact model results – we repeated the analysis using the mean and median value of the smaller cells for the aggregated cell with nearly identical results, but within-cell variance is lost through these methods. Some variables may aggregate better using variance measures instead. For example, slope values at 30 meters can rapidly lose meaning when aggregated due to variable terrain. Replacing a mean slope aggregate with a standard deviation of elevation among the smaller cells^55^ would better represent terrain ruggedness. It is also important to note that even though accuracy of models built at large grain sizes may remain high in most cases, there are many situations in which they will not be useful. The decision to model at a certain total extent and grain size thus should take into account research objectives (e.g., an estimation of a species range allowing a larger grain vs. a detailed understanding of environmental drivers of habitat suitability a finer grain), in addition to model accuracy.

Our analyses of predictive accuracy of models trained on the first three total extents replicated across the panda range suggest that the placement of a given study area on some range-wide environmental gradients drives both model accuracy and modifies the effect of total extent on model accuracy. Using percent forest cover as an example, the decrease in model accuracy with increasing forest cover makes sense due to the loss of the clear habitat suitability differences found between cells with more forest and those with less forest. This likely partially explains the decrease in accuracy we found along an increasing latitudinal gradient (Fig. S2), with models trained in the larger study extents within the Qinling mountains (Northeastern most part of the panda range) featuring more forest and lower predictive accuracies. Our finding that increasing environmental heterogeneity resulted in more accurate models follows expectations – higher variation in these variables should allow the models to better differentiate between suitable and unsuitable habitat^54^. The fact that we did not find a relationship between these increases in environmental heterogeneity and the effect of grain size on model accuracy was unexpected, however. This suggests that even grain sizes up to 1920 m capture enough of the environmental heterogeneity within study area replicates across the panda range to model habitat suitability with accuracy comparable to the finest grain sizes, regardless of the total level of environmental heterogeneity found within a given study area.

Recent developments in species distribution modeling beg more scale-related questions. The demonstration that MaxEnt is equivalent to an inhomogeneous Poisson process (IPP^12^) has resulted in the potential to incorporate the number of presence points within a cell in the modeling framework. The new open-source release of MaxEnt takes the IPP formulation and allows for the estimation of the relative abundance or probability of presence^56^. This has implications for the grain size used to represent the environment, as it replaces the binary presence/absence response with a count response which retains more information per cell as environmental data is aggregated to larger grain sizes. Specifically, as opposed to multiple occurrence points resulting in one presence cell, those points will be modeled as multiple observations of the species under the “same” environmental conditions. The relationship between the spatial autocorrelation of presence points^57^ and the grain size of environmental variables in both the original and IPP formulation of MaxEnt is another important scale-related question that future research should address. The same question would apply to any species distribution modeling framework in which the response variable could be defined as binary or as counts (generalized linear models and other regression frameworks).

The performance of models trained at increasingly larger extents when predicting panda distribution in the smallest extent yielded interesting results. The models trained at the smallest total extent were slightly outperformed by the models trained at the next-largest total extent, suggesting that there may have been environmental responses detected by the latter models that proved important when predicting at the smaller, nested total extent. The models trained at two largest total extent models performed much worse than smaller total extent models, however, indicating that there is a tradeoff between more fully capturing environmental responses and over-generalizing these responses when increasing total extent. Our visual analysis of the resulting suitability predictions of the smallest total extent highlighted the differences in their spatial patterns. Because predictions became more aggregated with increasing total extent and lost the detailed differences between high and low suitability areas, modeling at too large a total extent may have consequences for conclusions made about landscape connectivity. For example, the negative effects of roads are clear in the predictions of the models trained at the smaller total extents, but become increasingly smoothed with increases to total extent beyond 500 km^2^. This effect could result in researchers and managers to overestimate habitat connectivity across the landscape. The loss of fine-scale details in model predictions when using models built at a large total extent has been documented before^22,27^, but our study is the first to quantify the effect of increasing total extent on model accuracy across multiple total extents and present evidence that total extent can be optimized as larger than the area under investigation. Although previous research has indicated that total extent does not exhibit large effects on modeling landscape effects on connectivity^58^, our results have implications for modeling at landscape vs. local scales when attempting to model connectivity across high-disturbance areas. More investigation into these effects should be done with further testing of habitat models and movement data in the future.

A discussion of the effects of scale also informs knowledge on the ecology of giant pandas. In any ecological analysis of spatial scale, it is important to consider the relevance of the scales being analyzed to the species in question. As giant pandas generally use home ranges of around 5 km^2^, all of our total extents represented areas larger than a single panda would respond to, while our grain sizes varied from a maximum of about a single panda home range to a minimum of an area that would represent foraging habitat selection^59^. In terms of importance to the models, forest cover was relatively high at all total extents evaluated. Our finding that there was a generally positive effect of forest cover on habitat suitability at all extents and grain sizes (Fig. S9a) corroborates the widely accepted positive effect of forest on panda habitat in the literature^60,61^. That said, there has been recent evidence that pandas are able to utilize areas with bamboo but no forest cover^9^, and there were either neutral or even slightly negative suitability response curves to forest cover in a subset of replicates from all three smaller total extents that suggested this (Fig. S7).

The effects of roads on panda habitat suitability also varied across scale, and the decrease in importance of the distance to roads with increasing total extent suggests that the anthropogenic effects which accompany roads most strongly effect pandas at small scales. There was a high amount of variation in the suitability response to roads across the replicates of the smallest total extent, however (Fig. S8). Although most replicates featured models in which increasing distance to roads increased suitability, some featured models in which there was no response to roads and others saw a decreasing response. This likely reflects the large variation in traffic and roadside anthropogenic development that occurs across the panda range, and any positive road responses are likely due to more habitat availability in areas closer to roads. The expected negative responses to roads became more consistent in the models trained at increasing total extent sizes, confirming that positive responses are the exception.

The dominant variable in the models across most total extent/grain size combinations was elevation, which has long been accepted as important in modeling panda habitat^60,62–64^. Elevation itself is not directly important to panda habitat by itself, however, and is more of a proxy variable that relates with other factors such as climate variability^65^ (and in turn bamboo growth patterns) and anthropogenic disturbance. This is apparent in our study with the use of additional predictors like the five bioclimatic variables, which at the largest total extent reduced the importance of elevation from 60.7 to 15.3%. At the range-wide scale it was thus climatic factors that predicted habitat suitability better than elevation alone. At smaller total extents elevation likely represents the importance of local variations in climate due to topography and the importance it has on understory bamboo, while at larger total extents it is the regional variation in climate and both the physiological constraints and vegetation patterns^66^ that determine panda distribution. This is an example where although elevation is an effective variable for habitat prediction, its usefulness for explaining ecological phenomenon by itself is limited.

It is also important to consider the relevance and application to management when selecting different spatial scales. In the case of the giant panda, management occurs at total extents as small as parcels patrolled on foot, to entire nature reserves that approximate and sometimes exceed our 500 km^2^ extent^67^. Management also occurs at the provincial and national levels, approximating our third and largest total extents, respectively. This wide range in management hierarchies makes it important to consider the total extent at which panda distributions are modeled for a given question, which may not necessarily be better analyzed at the total extent of interest (e.g., our second-smallest total extent models best predicted the smallest total extent distributions while our second-largest total extent models were overall the most accurate when predicting to the total extent at which they were trained). We thus recommend, in addition to a *priori* consideration of scale, for researchers and managers to test for the effects of scale together with the use of additional variables on model performance. Optimizing the selection of spatial scale and variables in this manner will improve the use of species distribution modeling in both ecological research and wildlife management.

## Supplementary information

Supplementary information for “Interactive spatial scale effects on species distribution modeling: The case of the giant panda”.

## Data Availability

The environmental variables and R scripts used in the analysis will be deposited at https://issues.pangaea.de/browse/PDI-21776. Because of confidentiality agreements with government collaborators due to the threatened status of the giant panda and the sensitivity of their locational information, we are unable to make the 3^rd^ national giant panda survey location data publically available.
