# Supplementary material for: Interactive spatial scale effects on species distribution modeling: The case of the giant panda
Source: Sci Rep. 2019 Oct 10;9:14563. doi: 10.1038/s41598-019-50953-z (PMC6787011; doi:10.1038/s41598-019-50953-z)
Supplement: Supplementary file 1 — Supplementary information for “Interactive spatial scale effects on species distribution modeling: The case of the giant panda”. [file 41598_2019_50953_MOESM1_ESM.pdf]

Supplementary information for “Interactive spatial scale effects on species distribution modeling: The case of the giant panda”.

Thomas Connor, Andrés Viña, Julie Winkler, Vanessa Hull, Ying Tang, Ashton Shortridge, Hongbo Yang, Zhiqiang Zhao, Fang Wang, Jindong Zhang, Zejun Zhang, Caiquan Zhou, Wenke Bai, Jianguo Liu

This document contains eight supplementary figures complementing the main findings of the paper. Explanatory legends can be found under each figure. If a figure was too large to fit the legend on the same page, the legend can be found on the page previous to that figure.

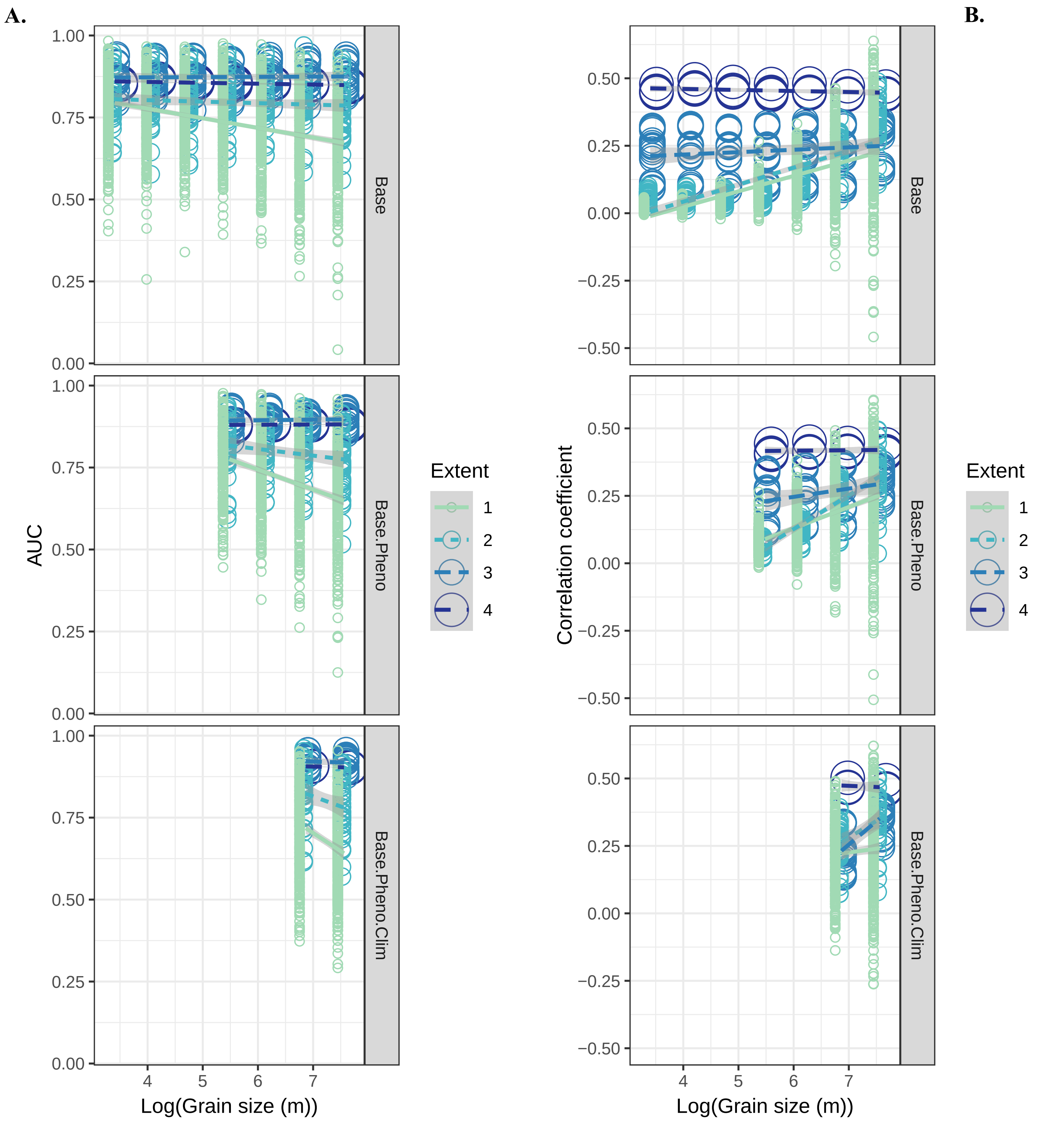

Figure S1: A. The effect of grain size on model AUC. B. The effect of grain size on the correlation between predicted probabilities of presence in test presences vs. test background points (cor).

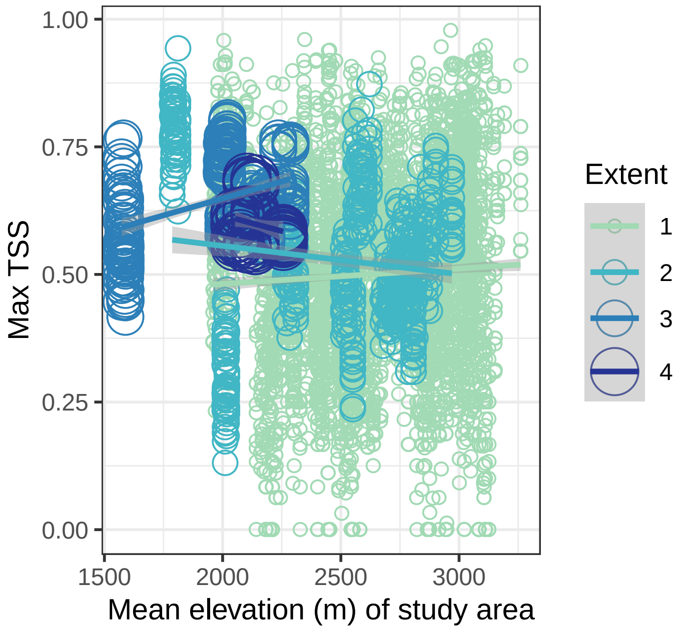

A.

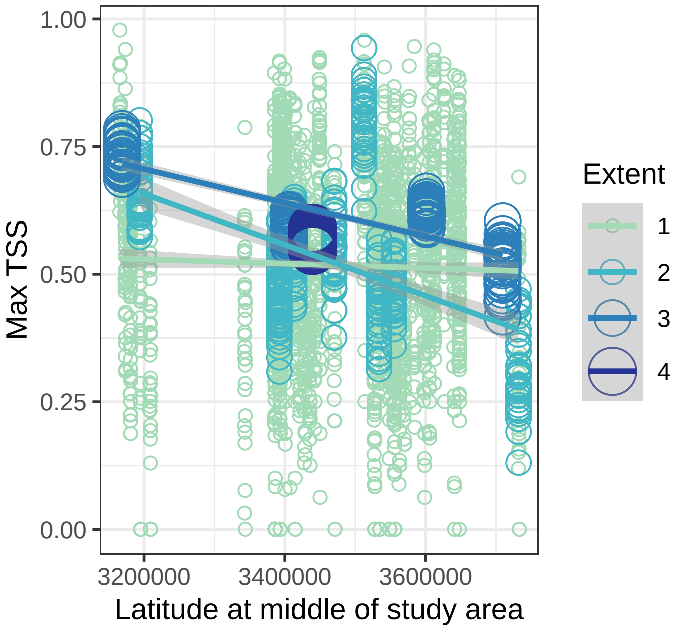

B.

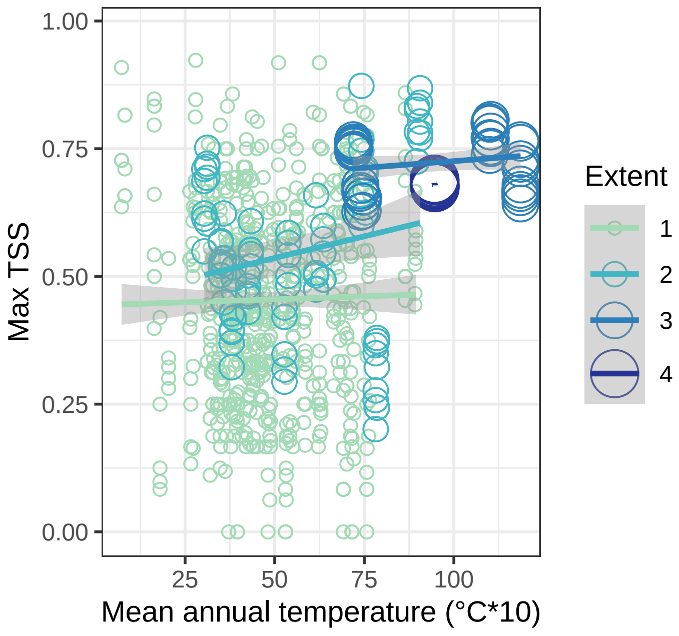

C.

Figure S2: The effect of the position of a study area along environmental gradients A-C on model accuracy as well as its impact on the effect of extent on model accuracy. A. Mean Elevation. B. Latitude. C. Mean of mean annual temperature.

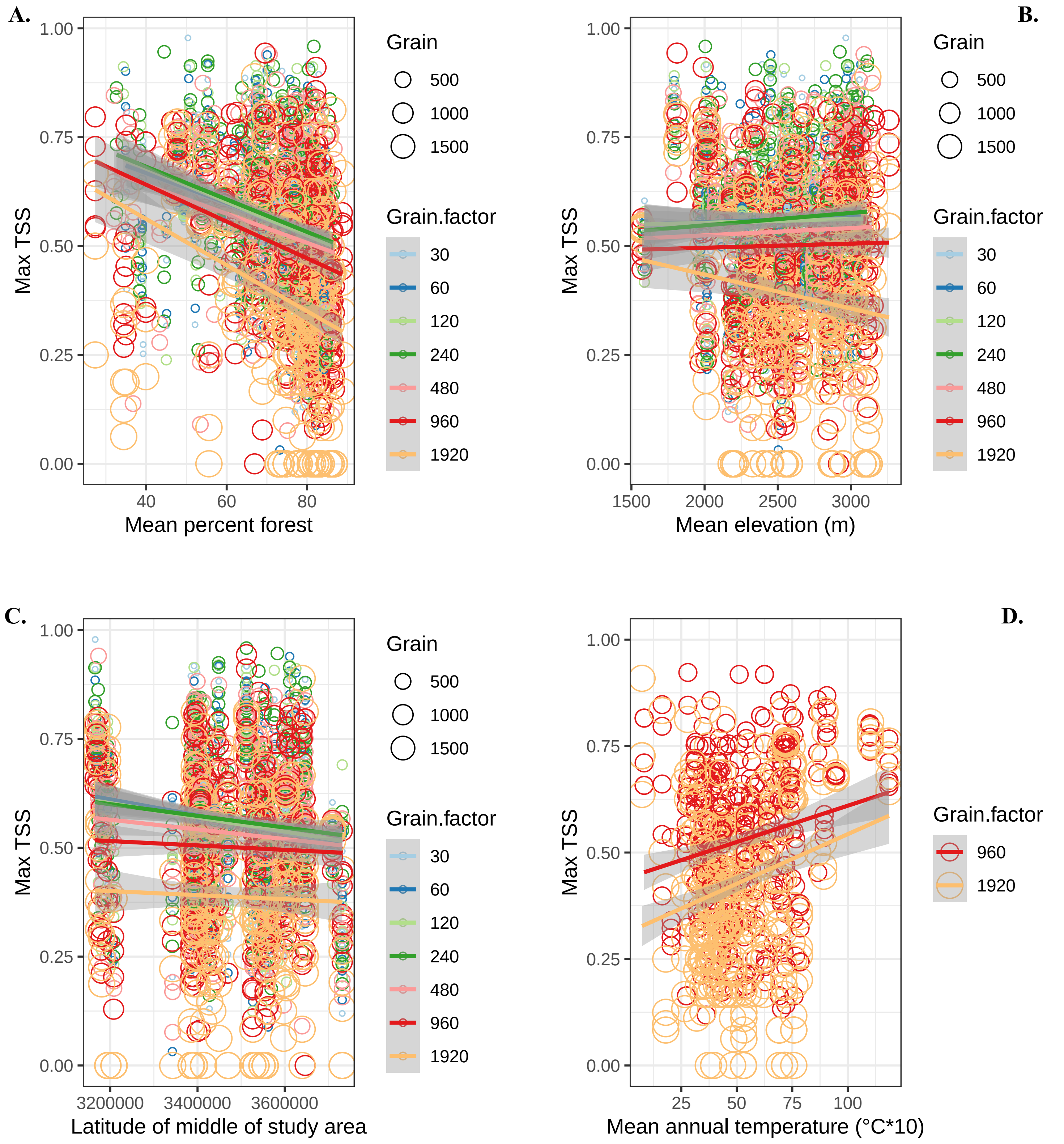

Figure S3: The effect of the position of a study area along environmental gradients A-D on model accuracy as well as its impact on the effect of grain size on model accuracy. A. Mean percent forest cover. B. Mean Elevation. C. Latitude. D. Mean of mean annual temperature.

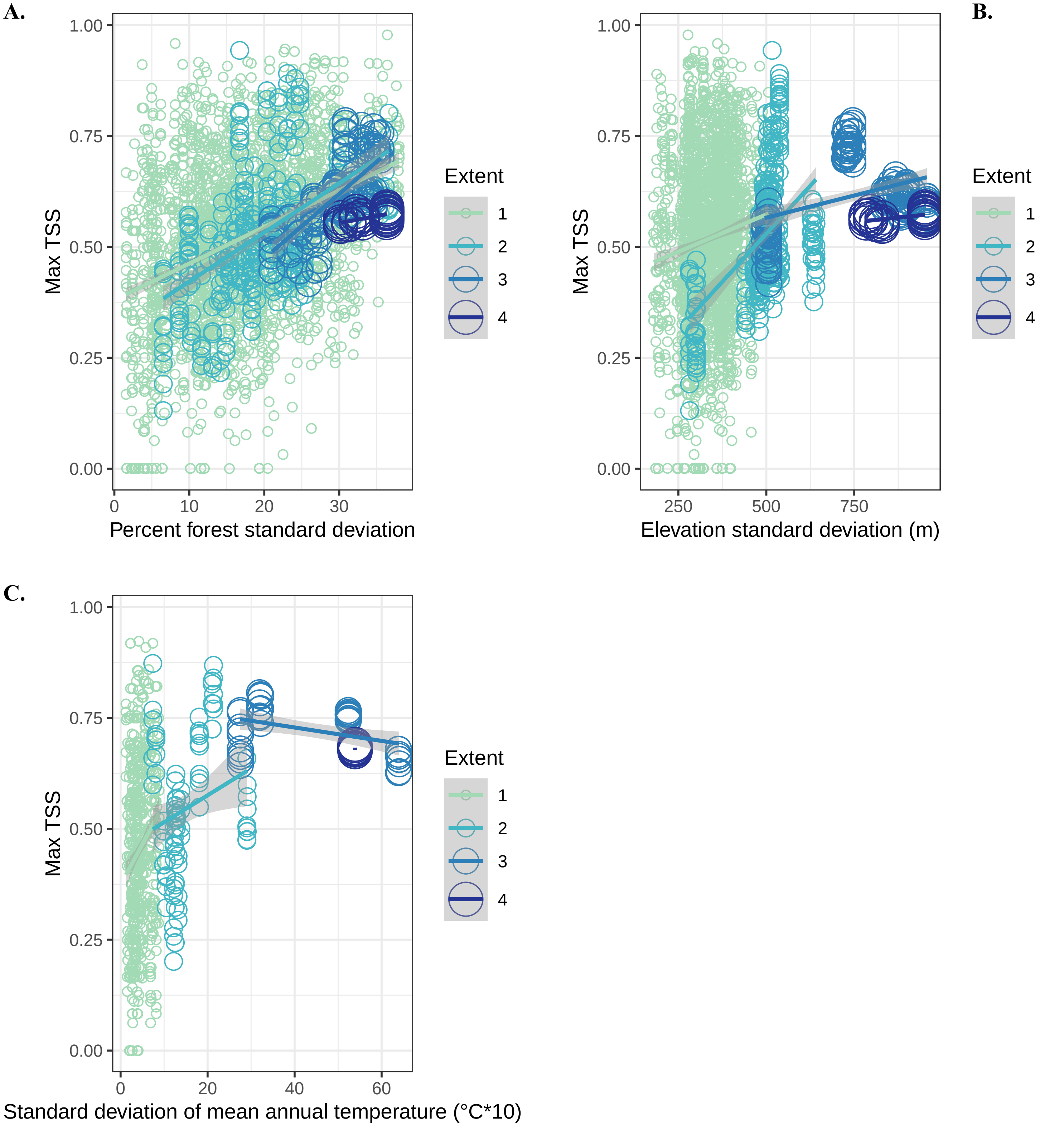

Figure S4: The effect of environmental heterogeneity within a study area on model accuracy as well as its impact on the effect of extent on model accuracy. A. Standard deviation of % forest cover. B. Standard deviation of elevation. C. Standard deviation of mean annual temperature.

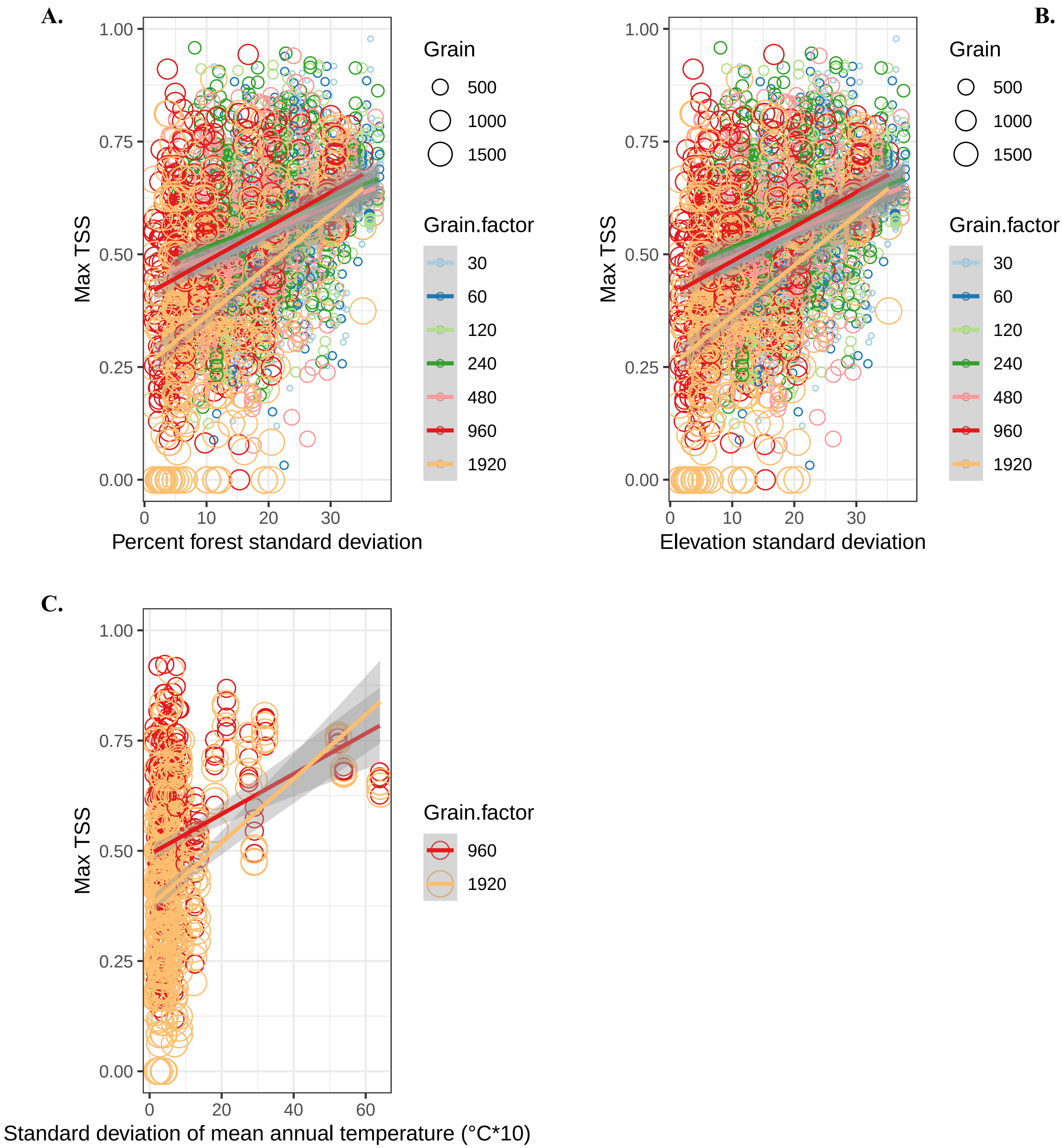

Figure S5: The effect of environmental heterogeneity within a study area on model accuracy as well as its impact on the effect of grain size on model accuracy. A. Standard deviation of % forest cover. B. Standard deviation of elevation. C. Standard deviation of mean annual temperature.

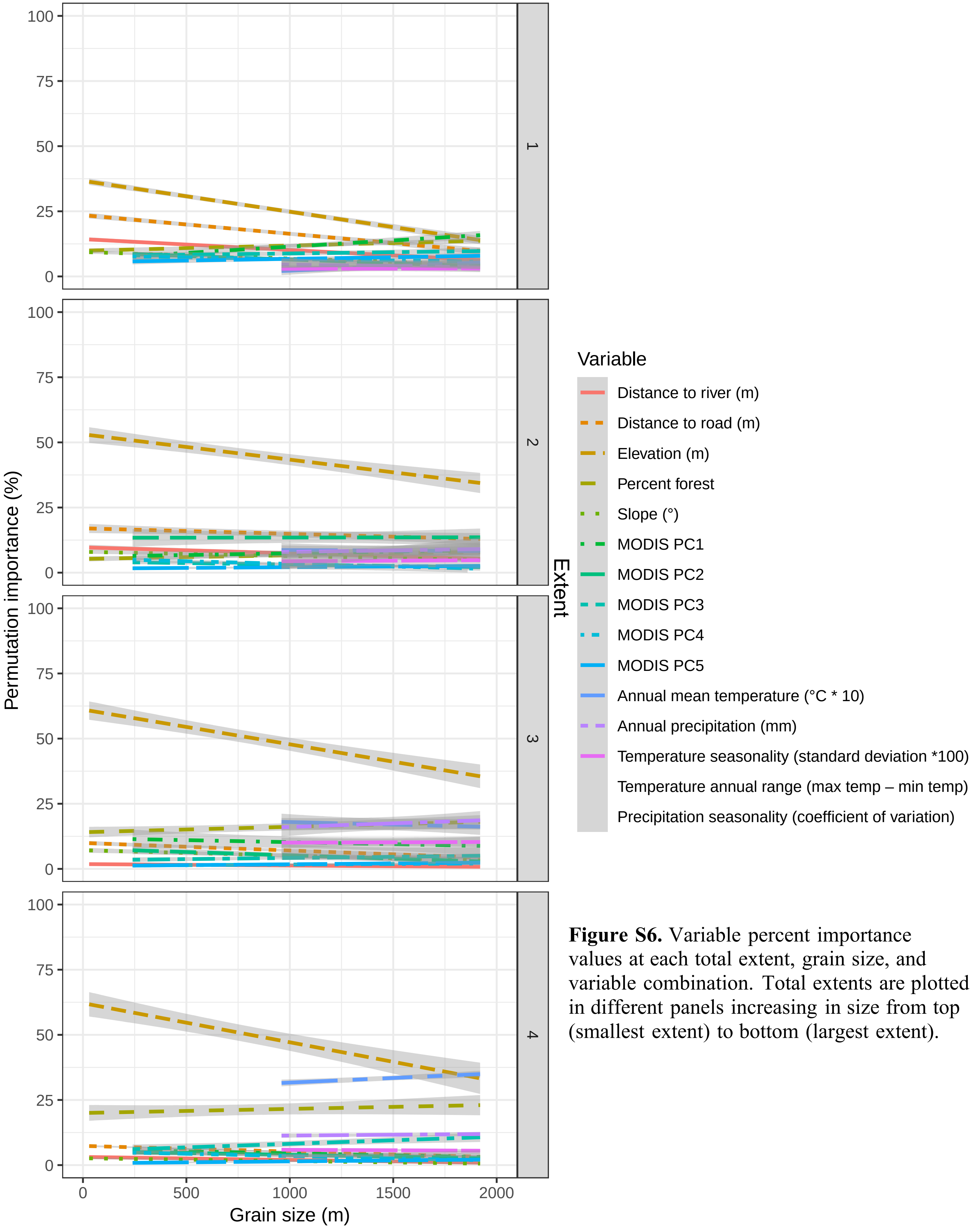

Figure S6. Variable percent importance values at each total extent, grain size, and variable combination. Total extents are plotted in different panels increasing in size from top (smallest extent) to bottom (largest extent).

Figure S7. The following pages contain environmental suitability response plots to percent forest cover (0-100%) derived from models trained with the base variable set at A. every total extent 1 replicate, B. every total extent 2 replicate, C. every total extent 3 replicate, and D. total extent 4.

Fig. 7A

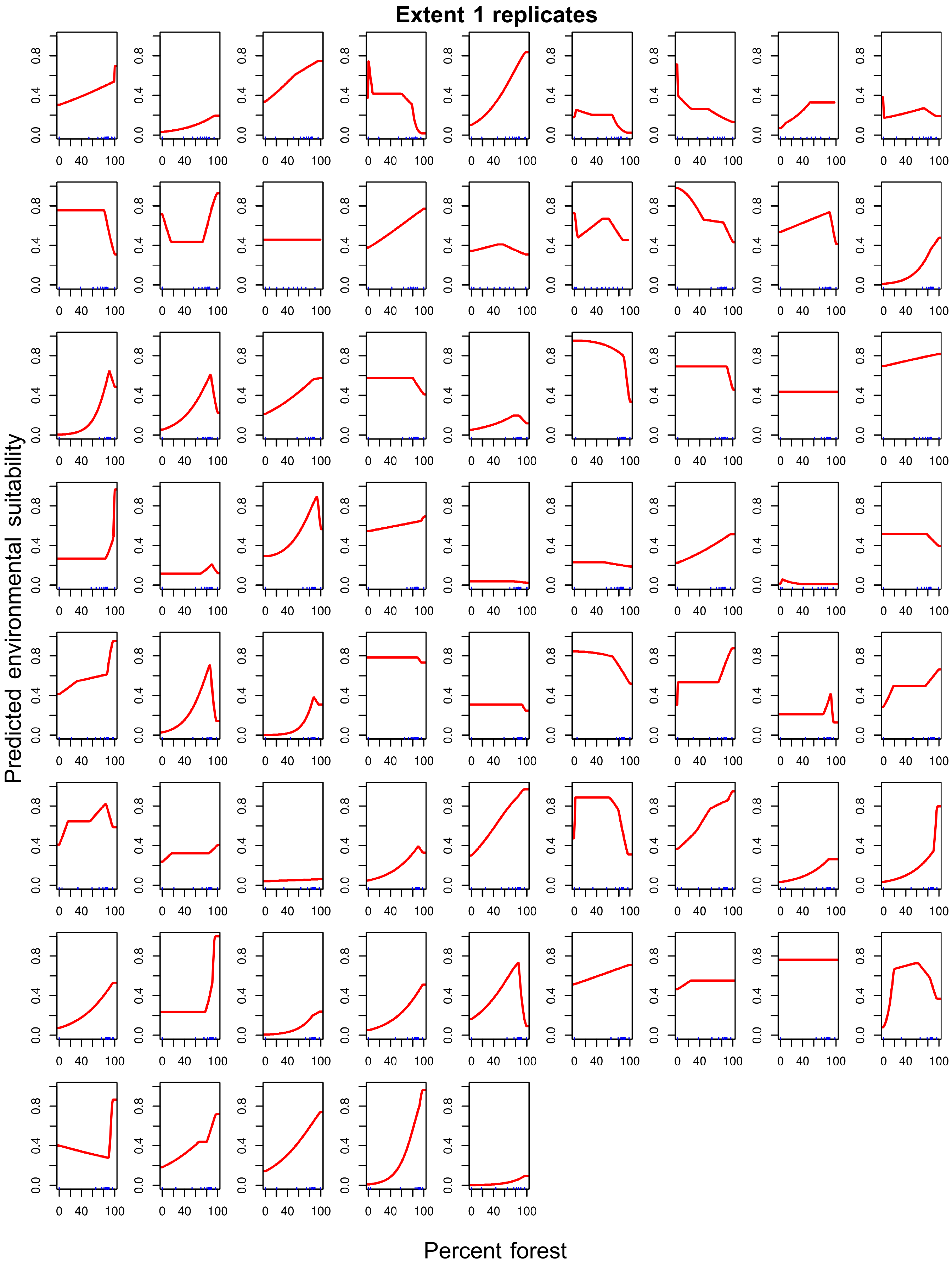
Extent 1 replicates
Predicted environmental suitability
Percent forest
0.0
0.4
0.8
0
40
100

Fig. S7B

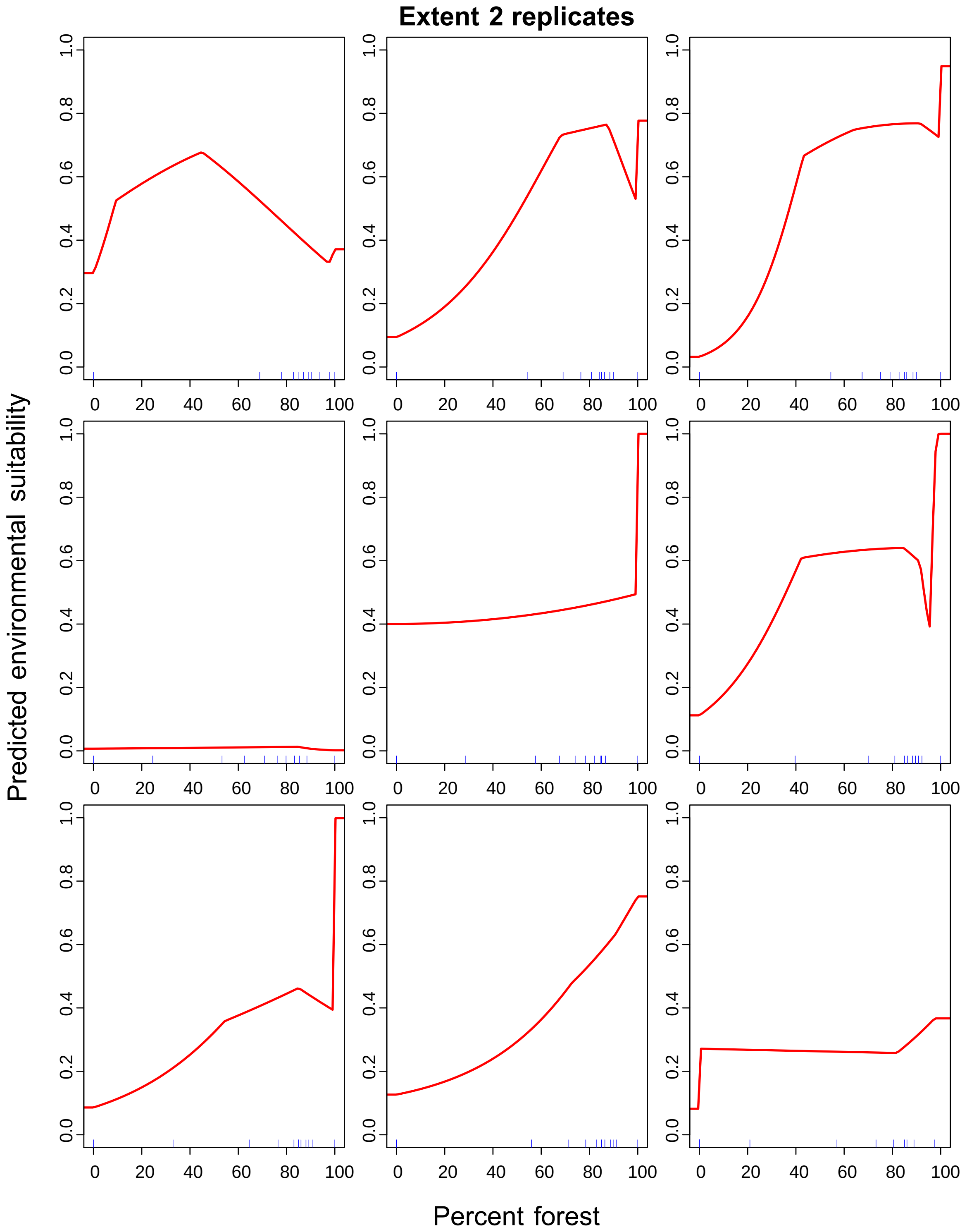

Fig. S7C

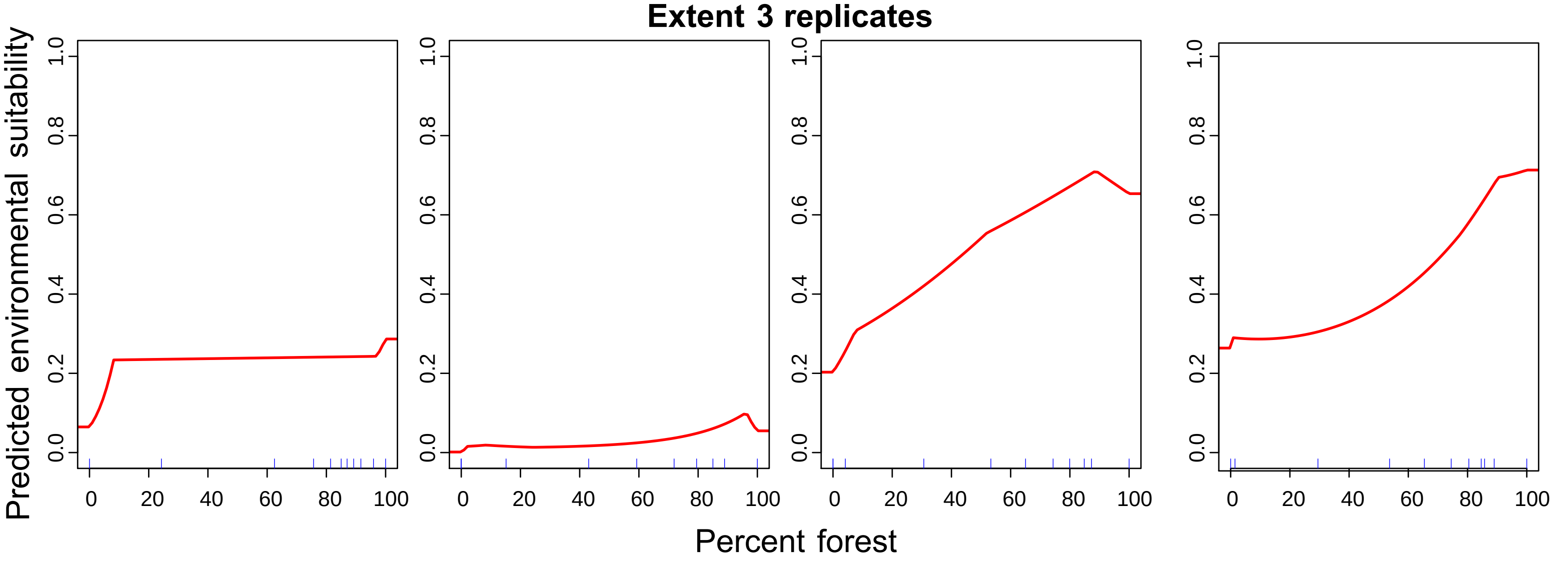

Fig. S7D

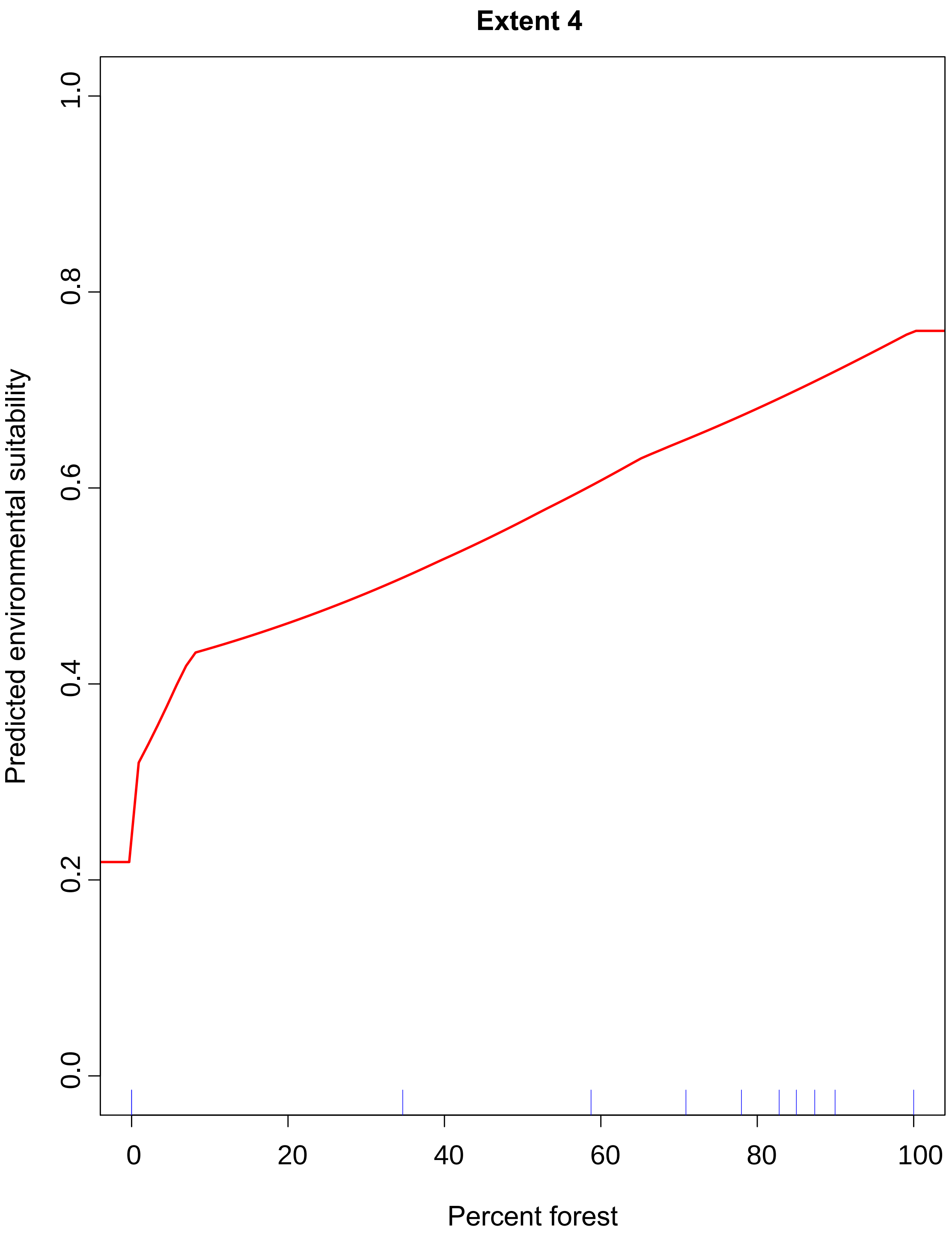

Extent 4
Predicted environmental suitability
0.0
0.2
0.4
0.6
0.8
1.0
0
20
40
60
80
100
Percent forest

Figure S8. The following pages contain environmental suitability response plots to increasing distances to roads (0-10000m) derived from models trained with the base variable set at A. every total extent 1 replicate, B. every total extent 2 replicate, C. every total extent 3 replicate, and D. total extent 4.

Fig. S8A

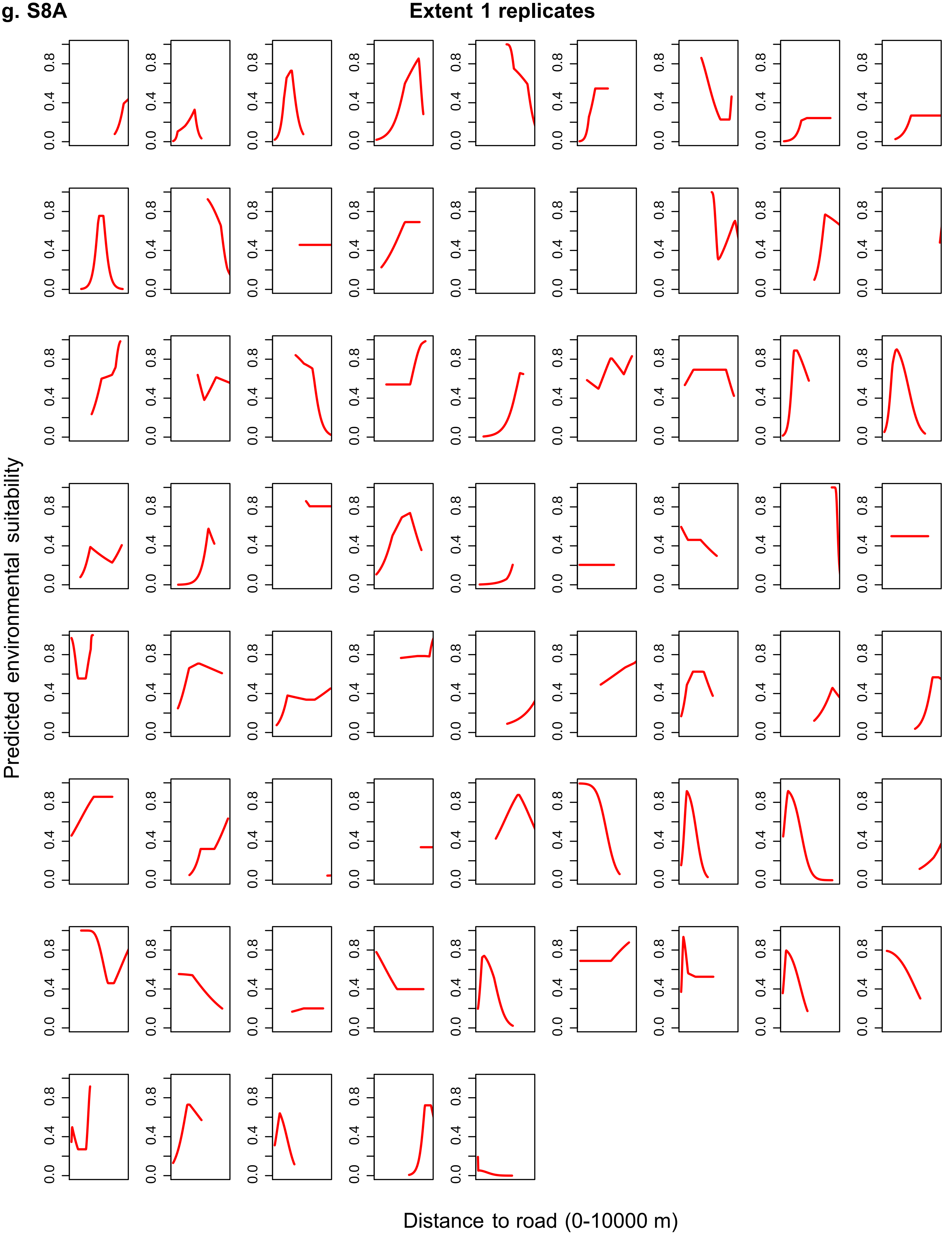
Extent 1 replicates
0.0
0.4
0.8
Predicted environmental suitability
Distance to road (0-10000 m)

Fig. S8B

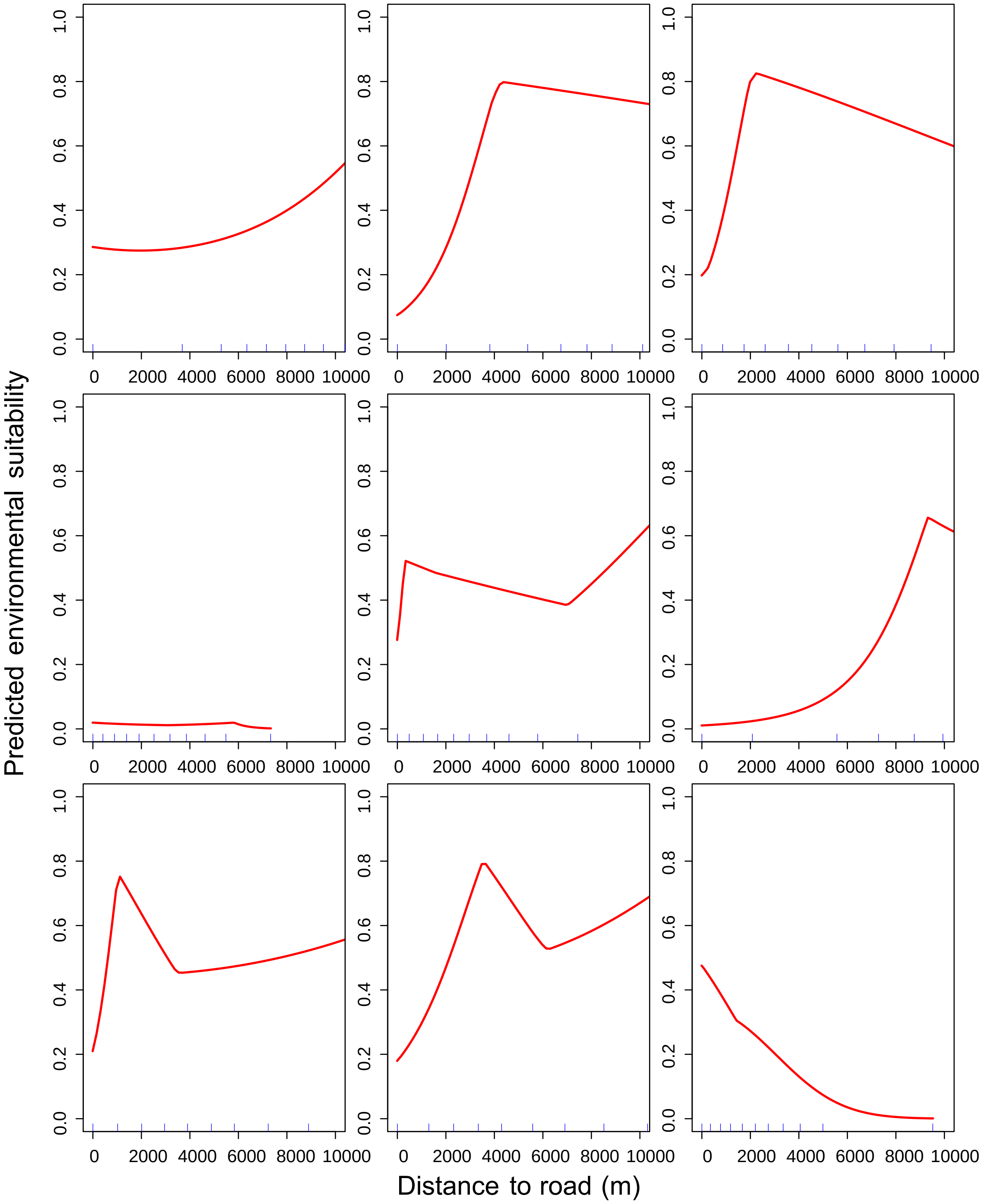

Fig. S8C

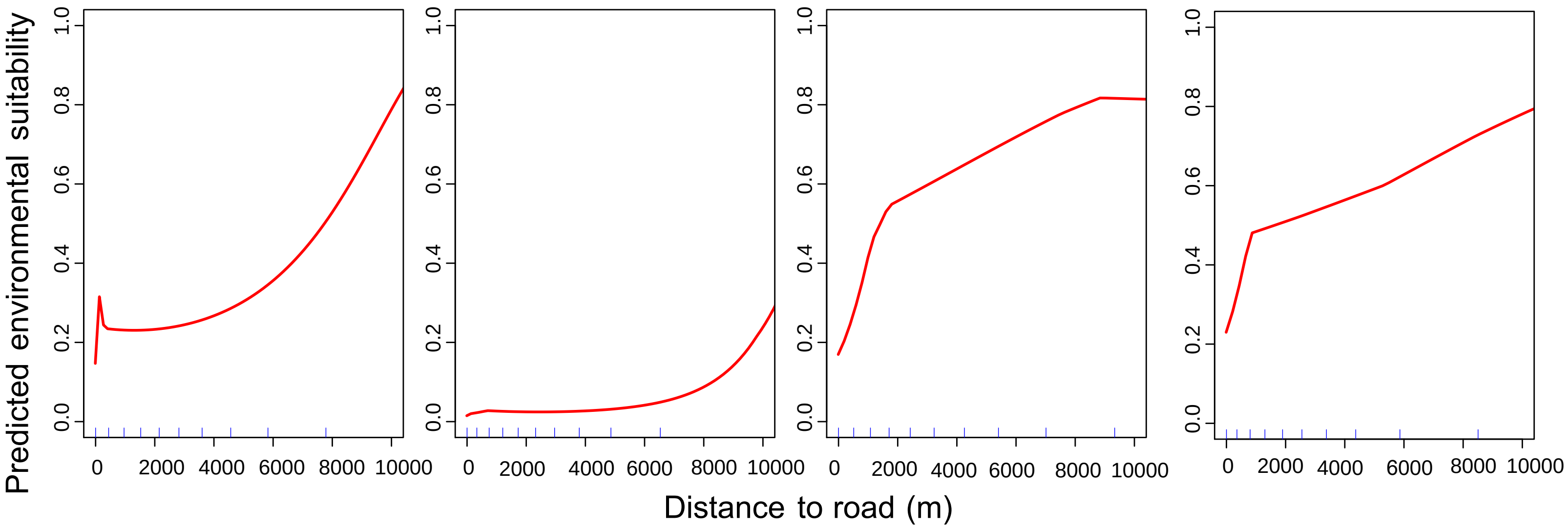

Fig. S8D

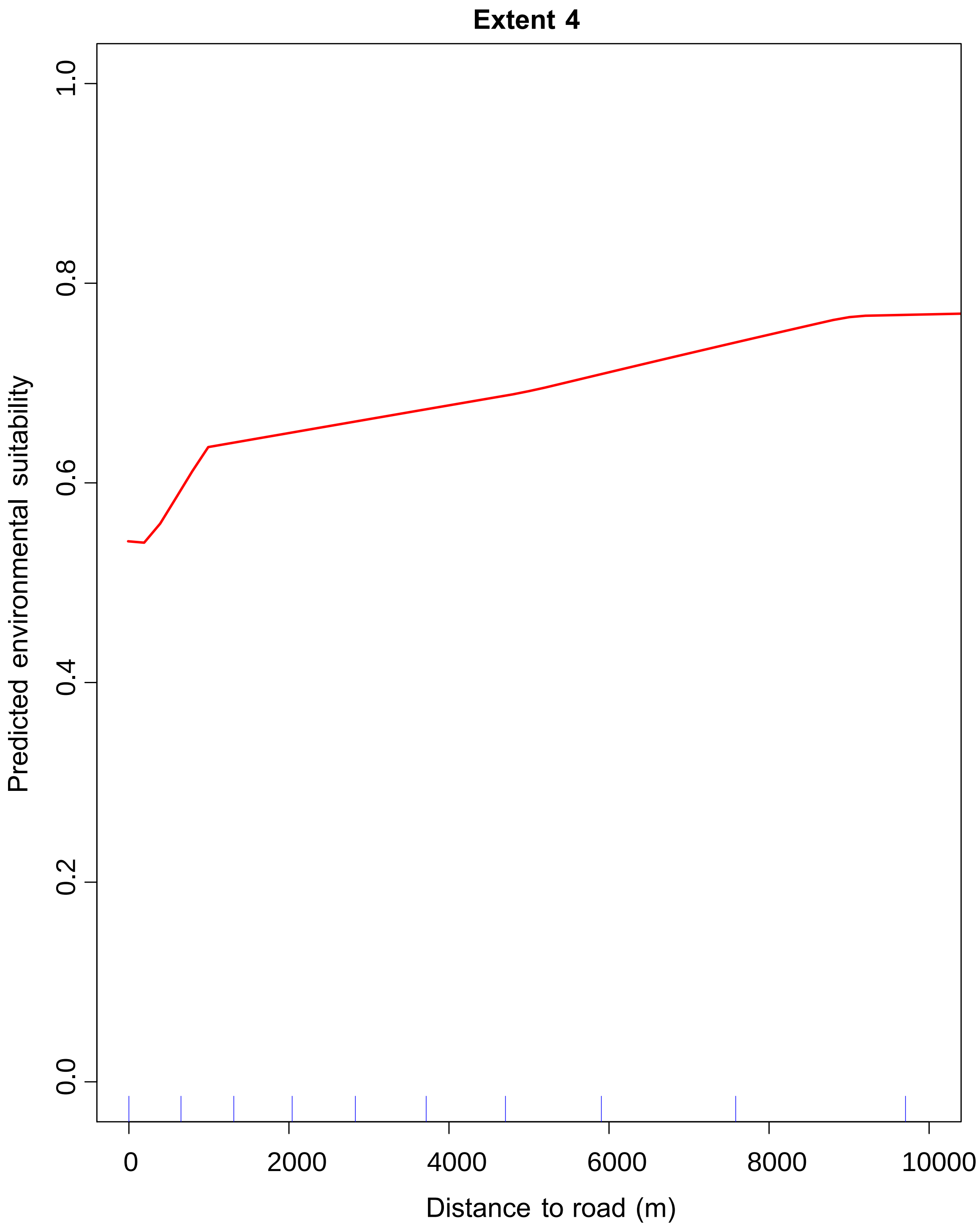
Extent 4
Predicted environmental suitability
0.0
0.2
0.4
0.6
0.8
1.0
0
2000
4000
6000
8000
10000
Distance to road (m)

Figure S9. The following pages contain environmental suitability response plots of models trained at increasing total extents and with increasing grain size to A. percent forest and B. elevation. Response plots are averages of every study area replicate at each total extent, and shaded areas are the 95% confidence interval around those averages.

Fig 9A

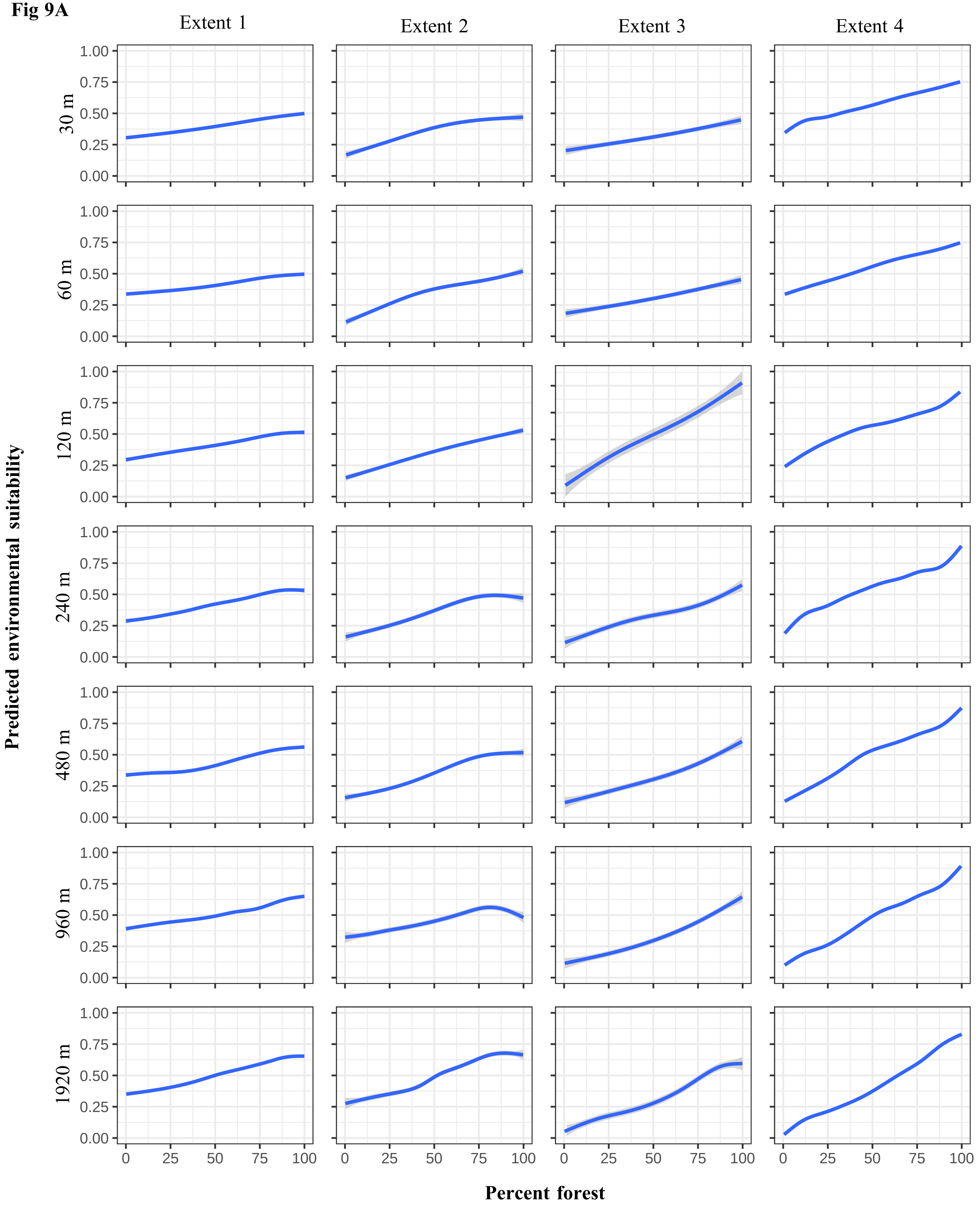

Extent 1
Extent 2
Extent 3
Extent 4
30 m
60 m
120 m
240 m
480 m
960 m
1920 m
1.00
0.75
0.50
0.25
0.00
0
25
50
75
100
Predicted environmental suitability
Percent forest

Fig. 9B

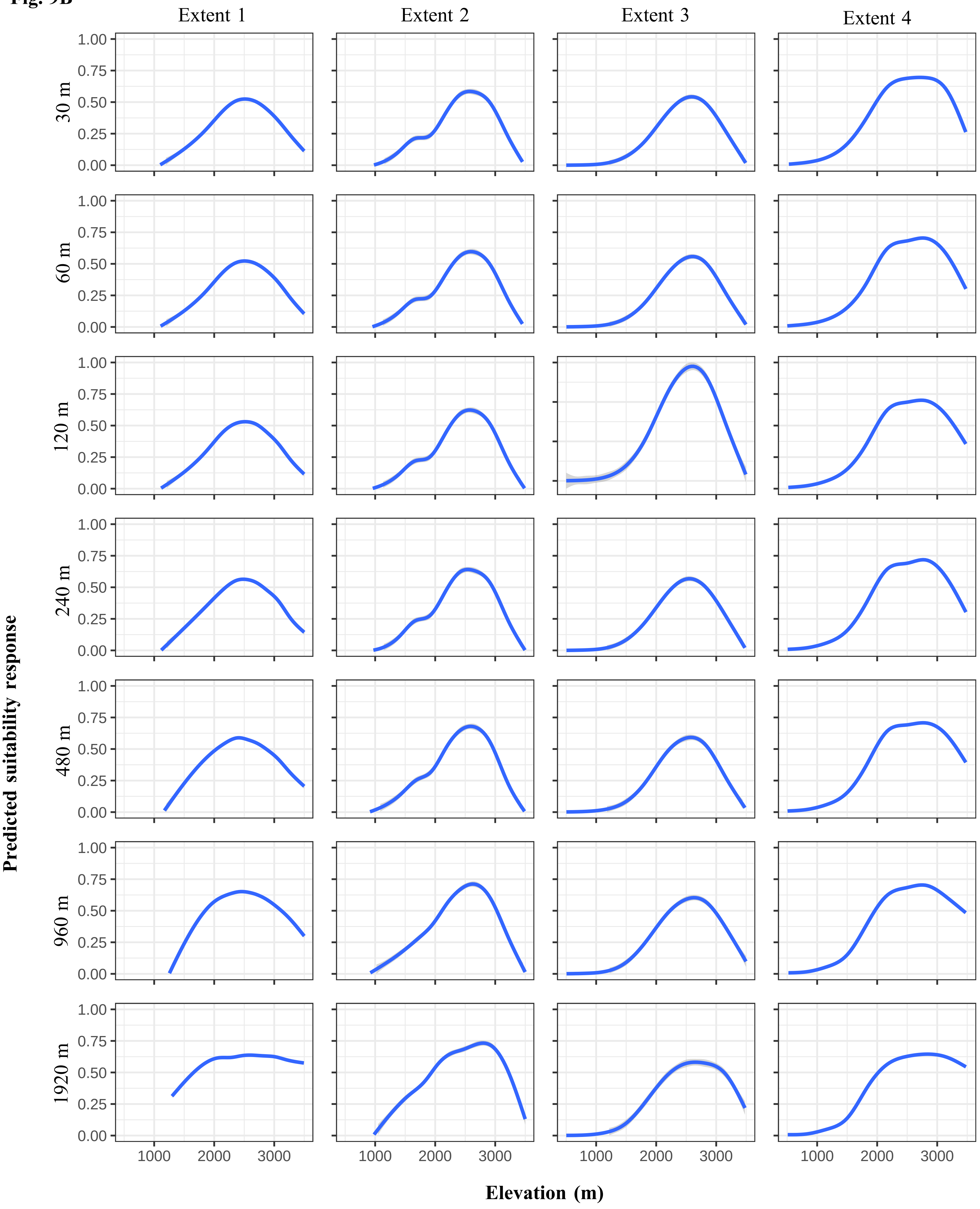
Extent 1
Extent 2
Extent 3
Extent 4
30 m
60 m
120 m
240 m
480 m
960 m
1920 m
1.00
0.75
0.50
0.25
0.00
1000
2000
3000
Predicted suitability response
Elevation (m)
